# Regioselective Dehydration of Sugar Thioacetals under Mild Conditions

**DOI:** 10.1021/acs.orglett.1c00424

**Published:** 2021-03-17

**Authors:** Rachel Szpara, Alexander Goyder, Michael J. Porter, Helen C. Hailes, Tom D. Sheppard

**Affiliations:** Department of Chemistry, Christopher Ingold Laboratories, University College London, 20 Gordon Street, London WC1H 0AJ, U.K.

## Abstract

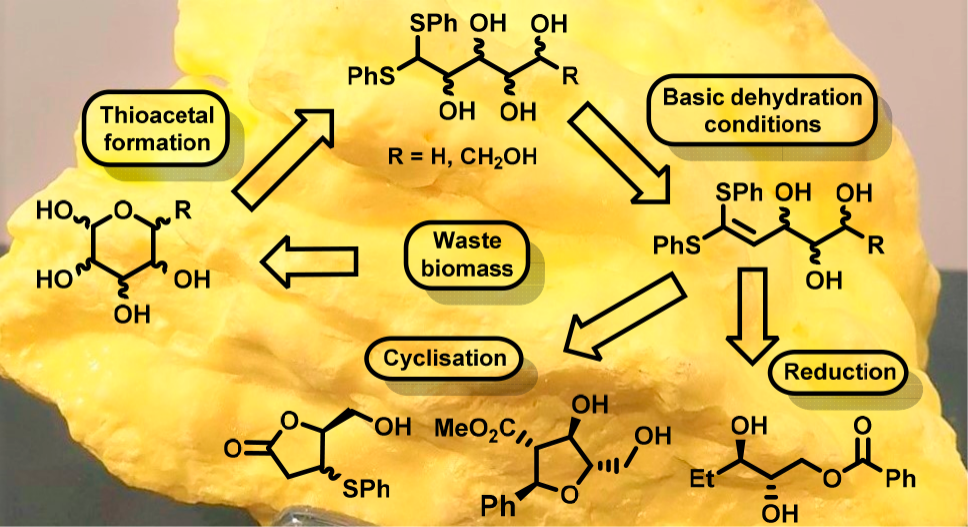

Sugars are abundant in waste biomass, making them sustainable chiral building blocks for organic synthesis. The demand for chiral saturated heterocyclic rings for pharmaceutical applications is increasing as they provide well-defined three-dimensional frameworks that show increased metabolic resistance. A range of sugar thioacetals can be dehydrated selectively at C-2 under mild basic conditions, and the resulting ketene thioacetals can be applied to the production of useful chiral building blocks via further selective dehydration reactions.

Carbohydrate biomass is an abundant renewable resource which has enormous potential for the synthesis of valuable chemical building blocks.^1^ The sugars present in this material are of particular interest as a functionalized carbon source to produce chiral saturated heterocycles which are of widespread potential utility in pharmaceutical development.^2^ While there are many well-established methods for converting sugars into chiral heterocycles such as tetrahydrofurans (THFs) and tetrahydropyrans (THPs), these typically rely on lengthy synthetic sequences involving the extensive use of protecting groups and high cost/energy reagents (e.g., Tf_2_O).^3^ They are therefore somewhat resource-intensive and relatively inefficient approaches, especially for the large-scale preparation of chiral building blocks, and chiral heterocycles derived from sugars remain relatively underexplored in drug discovery applications.^4^ The development of more efficient and sustainable synthetic routes to chiral building blocks from sugars is therefore of great interest, particularly if the use of protecting groups and high-cost reagents can be minimized or avoided. In this context, the identification of reactions that can be used to achieve the regioselective dehydration of sugars without the need for protecting groups is particularly important. Notably, the selective removal of one or more hydroxyl groups from the sugar backbone will lead to molecules with inherently more useful properties for pharmaceutical applications.

There have been recent reports of selective transformations of unprotected sugars and their derivatives using both biocatalytic^5−8^ and chemical approaches.^9−11^ Deoxygenation/dehydration of sugars is of particular interest, and only a few approaches have been described. For example, Gagné has reported methods for the regioselective reductive cyclization of protected sugar-derived polyols **1** using silane reagents^12,13^ in the presence of Lewis acids such as B(C_6_F_5_)_3_, leading to the formation of a range of chiral THFs and THPs **2** which can be accessed from sugars in a few steps (Scheme 1a).

**Scheme 1:**
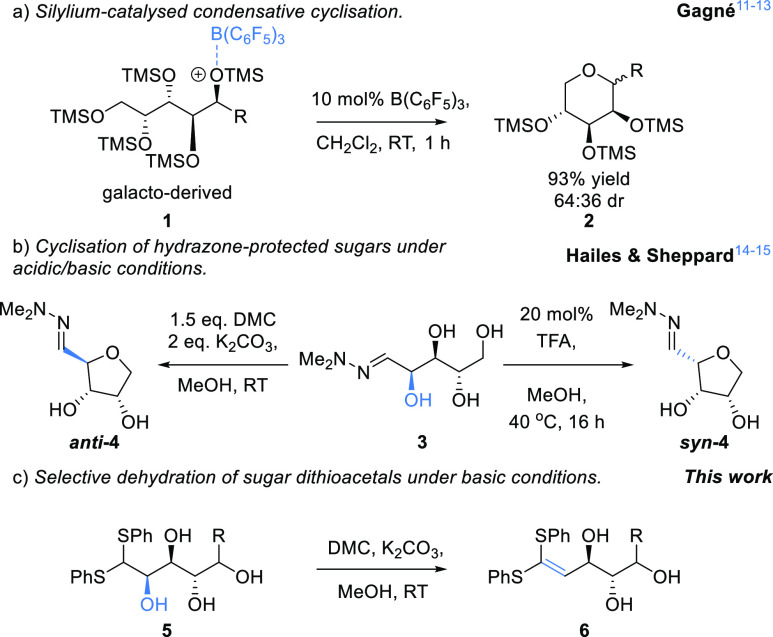
(a) Reductive cyclization of Silyl-Protected Sugars;^11−13^ (b) Chiral THF Formation via the Dehydration of Pentose Sugars;^14,15^ (c) This Work: Regioselective Dehydration of Sugar Thioacetals

In previous work, we have developed methods for the regioselective dehydration of sugar hydrazones, e.g., **3** (Scheme 1b), to give access to a range of chiral THFs (e.g., *syn*-**4** and *anti*-**4**) under very mild conditions.^14,15^ These reactions are readily scalable and provided access to useful chiral building blocks in only a few steps. Importantly, it was also observed that cyclization of the sugar hydrazones under acidic or basic conditions provides complementary stereoselectivities.^14^ The acid-catalyzed cyclization takes place under thermodynamic control, most likely proceeding via the stabilized diazenium cation, whereas the base-mediated cyclization appears to involve a kinetically controlled S_N_2 ring-opening of a cyclic carbonate intermediate which can epimerize prior to cyclization. In this latter reaction, it was rationalized that the main role of the hydrazone is to hold the sugar in the open-chain conformation which facilitates cyclization to the THF. We therefore envisaged that this approach could be extended to other open chain sugars such as thioacetals. Given that the formation of dimethylhydrazones from hexoses is often slow and relatively low-yielding, thioacetals might prove to be a more versatile alternative as they can readily be accessed from both pentoses and hexoses. In this paper, we describe methods for the regioselective dehydration of sugar thioacetals at C-2 and C-3 under mild and scalable conditions to provide access to novel chiral polyols and heterocycles (Scheme 1c).

Using l-arabinose, which is available from waste sugar beet pulp,^15,16^ as a test substrate, the corresponding ethyl and phenyl thioacetals were prepared via the reported procedures.^17,18^ Treatment of the ethyl thioacetal with K_2_CO_3_/dimethyl carbonate (DMC) led to the formation of a complex mixture of products. However, reaction of the readily formed phenyl thioacetal **5a**(18) under similar conditions led to the formation of the ketene thioacetal **6a** as a single product. In addition, purification of the phenyl thioacetal derivative could be achieved via recrystallization, avoiding the need for column chromatography. Interestingly, unlike the reactions of the corresponding hydrazones, the THF was not formed, and a selective dehydration took place exclusively at the C-2 position to give alkene **6a** in near-quantitative yield on a 5 g scale (Scheme 2).

**Scheme 2:**
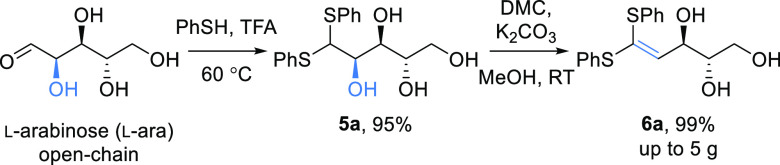
Thioacetal Protection of l-Arabinose Followed by Selective Dehydration under Mild Conditions^14,18^

The PhS groups in **5a** make the C-1 proton fairly acidic, and hence, it is clear that an elimination reaction can take place readily when the C-2 hydroxyl group is activated by DMC.^14^ The formation of similar ketene dithioacetals has previously been reported as a problematic side reaction in reactions of protected derivatives with strong bases (e.g., sodium methylsulfinylmethylide or *n*-BuLi).^19,20^ Given that our reaction conditions are very mild, and that the reaction is selective and high yielding, this potentially offers a readily scalable method for the selective C-2 deoxygenation of sugars without the need for hydroxyl protecting groups. The scope of this approach was then explored (Scheme 3). Selective dehydration was carried out with an array of sugar dithioacetals derived from aldose sugars, in moderate to excellent yields (48–99%) for several pentose and hexose sugars (**6a**, **6b**, **6e**, **6f**). However, some thioacetals, such as those derived from d-ribose (**5c**), l-rhamnose (**5d**) and d-mannose (**5g**), gave little to no conversion to the alkene. A common feature of the unsuccessful substrates is *anti*-stereochemistry at the C-2 and C-3 positions. This potentially provides a useful insight into the mechanism of the reaction, which is likely to occur via (reversible) formation of a cyclic carbonate at C-2/C-3, through reaction of the polyol with dimethyl carbonate. This then subsequently undergoes elimination by removal of the acidic C-1 proton (Scheme 4).

**Scheme 3:**
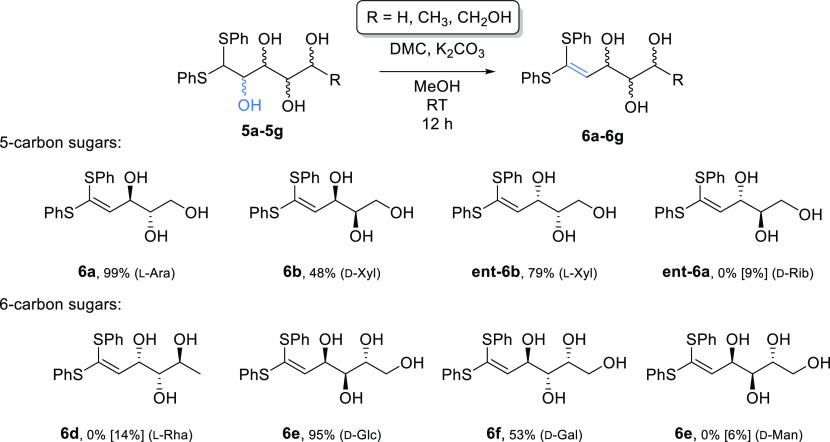
Selective Dehydration of Thioacetal-Protected Aldose Sugars at the C-2 Position under Basic Conditions Isolated yields. Conversions shown in brackets were determined by ^1^H NMR spectroscopy using an internal standard of 1,4-dimethoxybenzene.

**Scheme 4:**
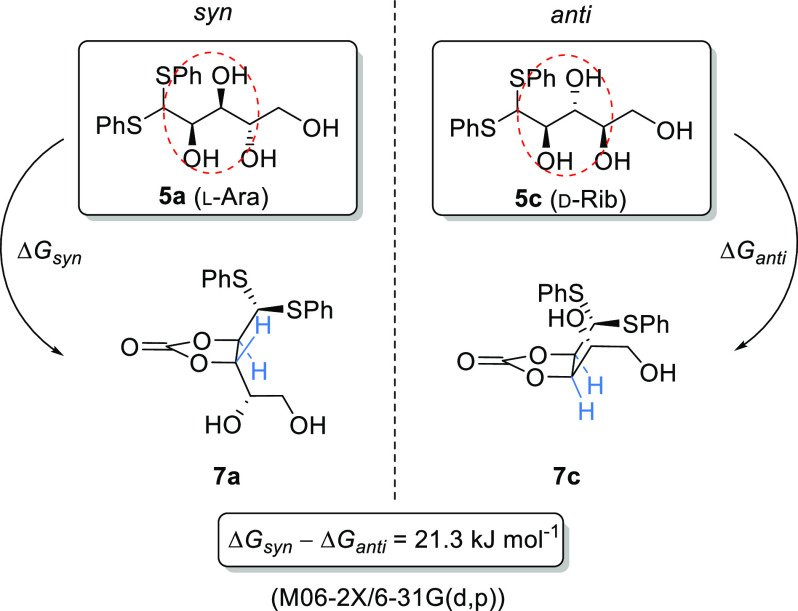
Proposed Carbonate Intermediates in the Dehydration Sugar Thioacetals under Basic Conditions DFT calculations suggest that the formation of a cyclic carbonate from a *syn* thioacetal **5a** is considerably less endergonic than from an *anti* sugar thioacetal **5c**.

The stereochemical relationship between the C-2 and C-3 alcohols may well affect the ease with which the carbonate can be formed (Scheme 4). As shown in structure **7c**, sugars with *anti* stereochemistry at C-2/C-3 (e.g., d-rib) will have to form the more sterically hindered *syn*-cyclic carbonate. This hindered carbonate may also disfavor alignment of the C-1 proton into the correct orientation for the subsequent E-2 elimination. In contrast, sugars with *syn*-stereochemistry at C-2/C-3 (l-ara) will form the less hindered *anti*-cyclic carbonate (e.g., **7a**) which can easily adopt the required conformation for E-2 elimination to generate the alkene. DFT calculations at the M06-2X/6-31G(d,p) level confirmed that the free energy change in going from **5a** to **7a** in methanol solution is ca. 21 kJ mol^–1^ more negative than that going from **5c** to **7c**.

Attempts to use more reactive electrophiles such as carbonyldiimidazole with **5c** failed to give any improvement in the yield, indicating that the stereochemical relationship in these starting materials presents a significant barrier to successful dehydration under mild reaction conditions. An alternative strategy was therefore considered for *anti*-sugars which did not rely on the formation of a cyclic intermediate. It was envisaged that conversion of the thioacetal **5c** to the corresponding peracetate could lead to sufficient activation of the C-2 alcohol for it to act as a leaving group, facilitating dehydration under basic conditions. Formation of the peracetate derivatives with pyridine/Ac_2_O,^21^ prior to treatment with a base was explored for the d-ribose, l-rhamnose, and d-mannose thioacetal derivatives (Scheme 5). Following acetylation, the protected sugars were stirred under basic conditions and monitored for ketene thioacetal formation. Although unreactive with K_2_CO_3_, the use of the stronger bases DBU (1,8-diazabicyclo[5.4.0]undec-7-ene), TBD (1,5,7-triazabicyclo[4.4.0]dec-5-ene), and ^t^BuOK led to formation of the desired products **8c**, **8d**, and **8g** in 47–96% yields. Different bases proved to be preferable for each example studied.

**Scheme 5:**
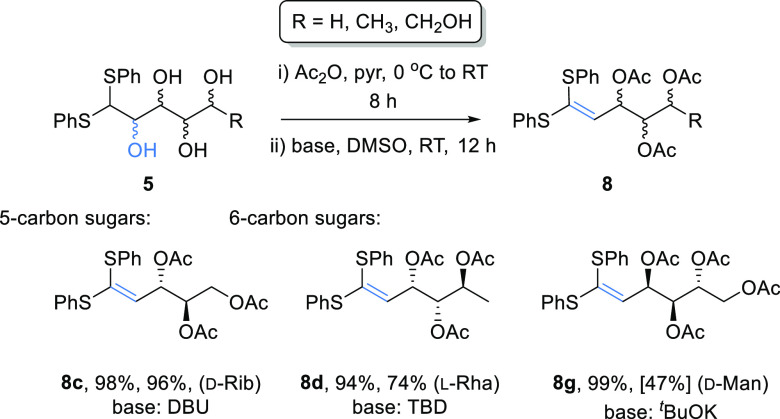
Selective Dehydration of the *Anti* Sugar Thioacetals via Initial Acetylation^21^ Followed by Base-Mediated Elimination Isolated yields are given for acetylation and dehydration steps, respectively. Conversion shown in brackets was determined by ^1^H NMR spectroscopy using an internal standard of 1,4-dimethoxybenzene.

With a series of sugar-derived ketene dithioacetals in hand, we then went on to explore the reactivity of these novel compounds (Scheme 6). We envisaged that reductive desulfurization of the ketene acetal group could lead to valuable chiral polyols containing a stereogenic center bearing an ethyl group. Thus, reduction of the l-arabinose derivative **6a** with Raney-Ni gave a triol **9**, which was isolated as the corresponding benzoate ester derivative **10** in 94% overall yield (Scheme 6). Depending on the sugar used, chiral polyols of this general structure could be useful in the synthesis of natural products such as eicosatetraenoic acid (precursor **11**),^22^ polysaccharides found in Gram-negative bacteria **12**,^23^ and cholesterol side-chains (dihydroxyvitamins).^24^ In principle, the alkene in **6a** has the potential to react with nucleophiles or electrophiles due to the ability of the two sulfur atoms to stabilize either an anion or a cation at C-1. However, it was not possible to observe any reactivity toward nucleophiles such as isopropylamine, morpholine, or sodium azide. Treatment of **6a** with an “activated” aldehyde equivalent (benzaldehyde dimethyl acetal) under Lewis acidic conditions at high dilution (0.03 M) (Scheme 6) was then explored in the hope that condensation of one of the hydroxyl groups would deliver the electrophile to the dithioalkene leading to an intramolecular ring-closure reaction. Pleasingly, this yielded the cyclized methyl ester **13** as a single diastereoisomer but in low yield (unoptimized). Ester **13** is presumably formed by trapping of the dithiolium cation with methanol followed by hydrolytic cleavage of the C–S bonds.

**Scheme 6:**
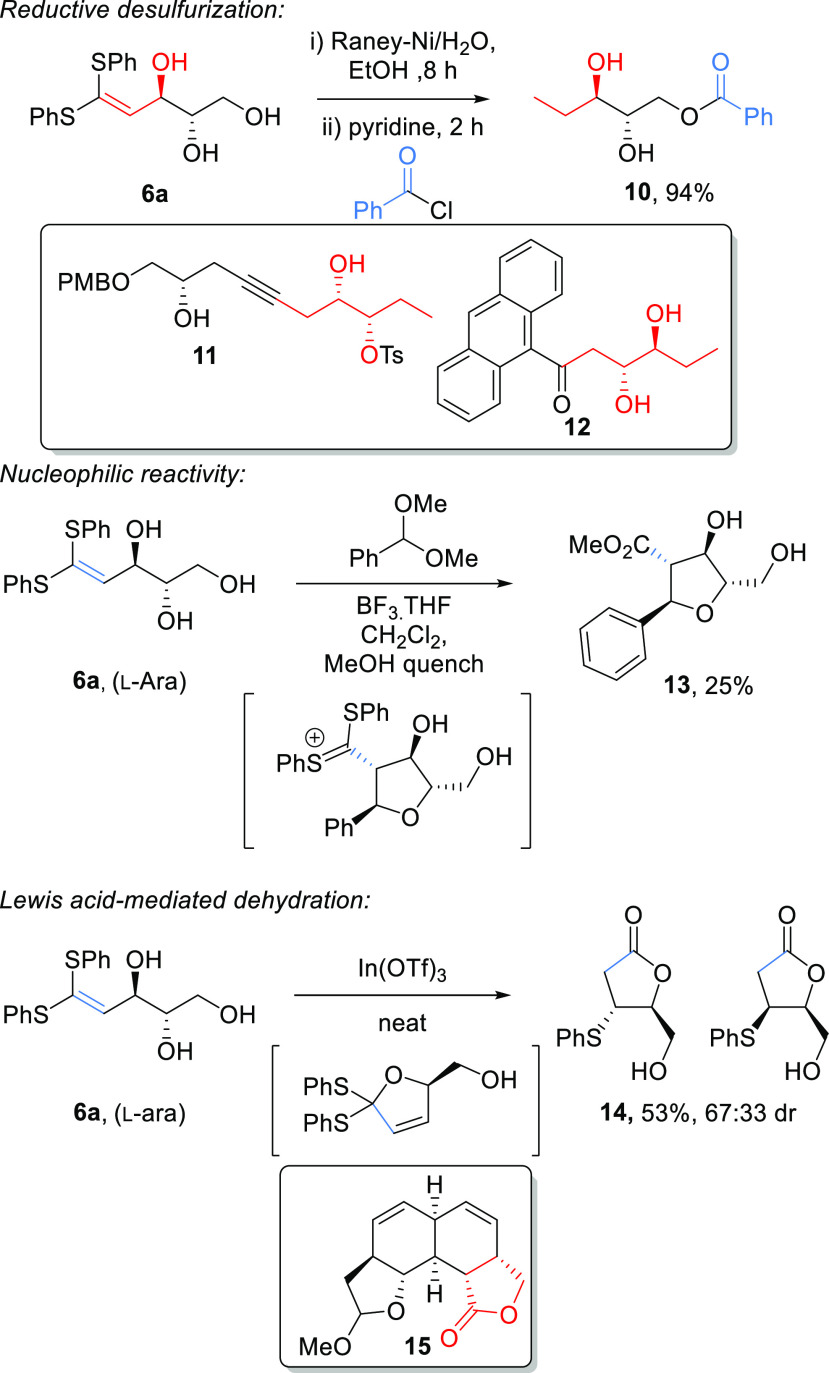
Further Reactions of l-Arabinose Ketene Thioacetal **6a** to Access Chiral Building Blocks Chiral motifs found in useful organic molecules are highlighted.

We also hypothesized that the allylic alcohol in ketene acetals **6** might be activated by the adjacent electron-rich alkene, making further selective dehydration at C-3 possible. Treatment of arabinose-derived thioalkene **6a** with In(OTf)_3_ led to cyclization at C-1, presumably via a stabilized allylic cation. This leads via hydrolysis to the α,β-unsaturated lactone which subsequently reacts with the liberated thiophenol to yield a diastereomeric mixture of known lactones **14** in 53% yield (unoptimized). Lactones **14** have been widely employed previously as building blocks for asymmetric synthesis^25,26^ directed toward natural products e.g. intermediate in Branimycin synthesis **15** (Scheme 6).^25^ Previously reported syntheses of lactones **14** are lengthy (6 steps) and required the use of harsh workup procedures and toxic solvents.^27^ In contrast, using our procedure, we were able to produce **14** in only three steps with recrystallization being the main method of purification.

In summary, we have developed scalable methods for the regioselective C-2 dehydration of sugar thioacetals.^28^ The resulting ketene thioacetals are versatile synthetic intermediates^29^ which can be used to access polyols containing a stereogenic center bearing an ethyl group. Preliminary studies have also demonstrated that further selective dehydration reactions and cyclization of these compounds can be used to access chiral heterocycles (THFs, butyrolactones) that are useful building blocks for asymmetric synthesis.

## Supporting Information Available

The Supporting Information is available free of charge on the ACS Publications Web site. Experimental procedures, details of DFT calculations including xyz coordinates for all calculated structures and ^1^H/^13^C NMR spectra for novel compounds. The Supporting Information is available free of charge at https://pubs.acs.org/doi/10.1021/acs.orglett.1c00424.

Experimental procedures, details of DFT calculations and ^1^H/^13^C NMR spectra for novel compounds (PDF)
*xyz* coordinates for all calculated structures (ZIP)
