# Supplementary material for: Regioselective Dehydration of Sugar Thioacetals under Mild Conditions
Source: Org Lett. 2021 Mar 17;23(7):2488–92. doi: 10.1021/acs.orglett.1c00424 (PMC8041386; doi:10.1021/acs.orglett.1c00424)
Supplement: Supplementary file 1 — ol1c00424_si_001.pdf [file ol1c00424_si_001.pdf]

# Supplementary Information

## Regioselective Dehydration of Sugar Thioacetals under Mild Conditions

Rachel Szpara, Alexander Goyder, Michael J. Porter, Helen C. Hailes and Tom D. Sheppard*

[a]Department of Chemistry, Christopher Ingold Laboratories, University College London, 20 Gordon Street, London WC1H 0AJ, U.K
tom.sheppard@ucl.ac.uk

# Table of Contents

Experimental .......... 3
General Experimental .......... 3
Experimental Procedures and Data .......... 4
DFT Calculations .......... 16
References .......... 18
Spectra: $^{1}H$ & $^{13}C$ NMR: .......... 19

# Experimental

## General Experimental

All reagents and solvents were purchased from Sigma Aldrich, Fisher Scientific, Fluorochem and Acros Organics and used as supplied unless otherwise noted. All reactions were monitored by TLC or $^{1}$H NMR. TLC analysis was conducted using aluminium plates pre-coated with silica gel 60 F254 (Merck KGaA). The spotted TLCs were visualised by UV light at 254 nm or appopriate staining agents. Reacions were heated using aluminium heating blocks. Column chromatography purification was performed using a Biotage Isolera flash purification system with Buchi FlashPure flash cartridges prepacked with silica gel (40-60 µm). Petrol mentioned in procedures is petroleum ether b.p = 40-60 °C. $^{1}$H NMR and $^{13}$C NMR spectra were recorded at 400, 500, 600 or 700 MHz (for $^{1}$H) and 151 or 176 MHz (for $^{13}$C) on Bruker AMX400, AMX500, AMX600 spectrometers at ambient temperature, unless otherwise indicated. Peaks are assigned as singlet (s), doublet (d), triplet (t), quartet (q), quintet (qn), or multiplet (m). All shifts are reported in parts per million (ppm) and compared against residual solvent signals: $CHCl_3$ ( = 7.26 ppm, s), MeOH ( = 3.31 ppm, qn) or DMSO ( = 2.50 ppm, qn) as the internal standard. Coupling constants (*J*) are quoted in Hertz (Hz) to one decimal place. Mass spectrometry was performed by the UCL Chemistry Mass Spectrometry Facility using either a Agilent LC- 6510 Q-TOF mass spectrometer (ThermoFischer Scientific) with a TOF mass analyzer or the Orbitrap™ Q Exactive mass spectrometer with a FTMS mass analyzer (ES+, CI, ES- modes). Infrared spectra were obtained using a Perkin-Elmer Spectrum 100 FTIR Spectrometer operating in ATR mode, all frequencies given in reciprocal centimetres (cm $^{-1}$). Melting points were measured with a Gallenkamp heating block and are uncorrected. Optical rotation was measured using an AA-65 Optical Activity Ltd Polarimeter with working wavelength = 589.44 nm, all $[\alpha]_D$ values were obtained using 10 mg/mL solutions unless otherwise stated, and are given in degrees cm $^{3}$g $^{-1}$ dm $^{-1}$.

## Experimental Procedures and Data

### (2*S*,3*S*,4*R*)-5,5-bis(phenylthio)pentane-1,2,3,4-tetraol [5a]:[1]

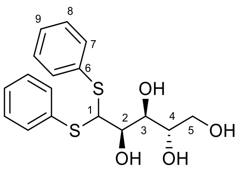


A mixture of L-arabinose (9.0 g, 60.0 mmol) and thiophenol (13.3 mL, 130 mmol, 2.2 eq.) in a mixture of trifluoroacetic acid and water (9:1, 5.0 mL) was stirred at 50-60 °C for 12 h. The reaction solution was then concentrated *in vacuo* to a solid residue, which was then recrystallised from boiling EtOAc before washing with $Et_2O$ (2 × 10 mL) to yield the desired thioacetal (19.7 g, 56.0 mmol, 95%): m.p. = 180-183 °C (EtOAc) [183-186 °C][1]; $[\alpha]_D^{21}$ = −20 (*c* = 1.00, pyridine) [−23, *c* = 1.10, pyridine][1]; $^1$H NMR (700 MHz, MeOD) $\delta$ 7.46-7.42 (m, 4H, H-7), 7.31-7.20 (m, 6H, H-8, H-9), 4.76 (d, *J* = 7.7 Hz, 1H, H-1), 4.12 (d, *J* = 2.1 Hz, 1H, H-3), 4.01 (dd, *J* = 7.7, 2.1 Hz, 1H, H-2), 3.78 (d, *J* = 11.2 Hz, 1H, H-5), 3.70-3.65 (m, 1H, H-4), 3.62 (d, *J* = 11.2 Hz, 1H, H-5); $^{13}$C NMR (176 MHz, MeOD) $\delta$ 136.8 (Ar-C), 133.8 (Ar-C), 129.7 (Ar-C), 128.4 (Ar-C), 73.2 (C-2), 72.8 (C-3), 72.0 (C-4), 64.9 (C-5), 64.5 (C-1); $\nu_{max}$ (solid/cm$^{-1}$) 3300 (O-H), 1640 (Ar C=C), 1437 ($CH_2$), 1085 (C-O). Data in accordance with the literature.[1]

### (2*R*,3*S*,4*R*)-5,5-bis(phenylthio)pentane-1,2,3,4-tetraol [5b]:[1]

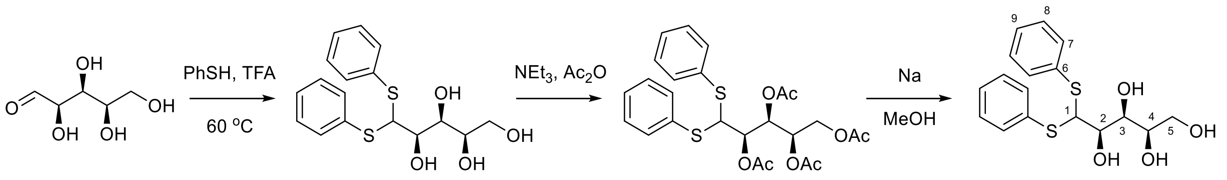


**pre-5b**

A mixture of D-xylose (3.00 g, 20.0 mmol) and thiophenol (4.51 mL, 40.0 mmol, 2.2 eq.) in 90% trifluoroacetic acid in water (3.00 mL) was stirred at 50-60 °C 12 h. The reaction solution was then concentrated *in vacuo* to give the crude xylose diphenyldithioacetal. A solution of the crude xylose diphenyl thioacetal (8.7 g, 24.0 mmol) in $NEt_3$ (48 mL, 0.34 mol) and acetic anhydride (16 mL, 0.17 mol) was stirred at room temperature for 12 h. The mixture was then poured onto ice water (600 mL). After 15 min, the mixture was extracted into $CH_2Cl_2$ (2 × 50 mL) and the combined extracts were dried over $MgSO_4$ before concentrating *in vacuo*. A solution of the product in $CH_2Cl_2$ was filtered through a pad of silica, washing with $CH_2Cl_2$. Evaporation of the filtrate and crystallisation of the product from a 4:1 ether/petrol mixture (50 mL) at 0 °C gave a white solid as a crude product. The crude material was then purified via silica gel chromatography (2:1, EtOAc:petrol) to give D-xylose diphenyl thioacetal tetraacetate **pre-5b** (959 mg, 160.0 mmol, 19%).[2]

A solution of **pre-5b** (959 mg, 160 mmol) and sodium (10 mg) in methanol (50 mL) was left to stir for 6 h, followed by addition of dry ice (2 g) and subsquent filtration. The

filtrate was evaporated to leave a residue, trituration of this solid with ethanol caused it to crystallise to an orange waxy solid (482 mg, 1.37 mmol, 86% crude). Recrystallisation from hot methanol gave the product as fine white crystals (134 mg, 0.38 mmol, 24%): m.p. = 113-118 °C (EtOH) [100-101 °C][1]; $[\alpha]_D^{21}$ = −12 ($c$ = 1.00, pyridine) [−8, $c$ = 1.00, pyridine][1]; $^1$H NMR (600 MHz, MeOD) $\delta$ 7.45-7.39 (m, 4H, H-7), 7.32-7.20 (m, 6H, H-8, H-9), 4.74 (d, $J$ = 5.0 Hz, 1H, H-1), 4.15-4.10 (m, 2H, H-5), 4.07 (m, 1H, H-3), 3.97 (t, $J$ = 5.0 Hz, 1H, H-2), 3.88 (m, 1H, H-4); $^{13}$C NMR (151 MHz, MeOD) $\delta$ 136.0 (Ar-C), 133.2 (Ar-C), 130.2 (Ar-C), 128.8 (Ar-C), 74.5 (C-2), 72.8 (C-3), 71.7 (C-4), 66.9 (C-5), 64.3 (C-1); $\nu_{\max}$ (solid/cm$^{-1}$) 3216 (O-H), 1578 (CH), 1045 (C-O). Data in accordance with the literature.[1]

**(2*S*,3*R*,4*S*)-5,5-bis(phenylthio)pentane-1,2,3,4-tetraol [*ent*-5b]:**

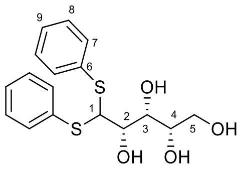


A mixture of L-xylose (3.0 g, 20.0 mmol) and thiophenol (4.5 mL, 40.0 mmol, 2.2 eq.) in 90% trifluoroacetic acid in water (5.0 mL) was stirred at 50-60 °C for 12 h. The reaction solution was then concentrated *in vacuo* to a solid residue, which was then recrystallised from $CH_2Cl_2$ before washing with $Et_2O$ (2 × 10 mL) and drying *in vacuo* to yield the product as a fine white powder (1.32 g, 3.7 mmol, 20%): m.p. = 111–114 °C ($CH_2Cl_2$); $[\alpha]_D^{21}$ = +12 ($c$ = 1.00, pyridine); $^1$H NMR (700 MHz, MeOD) $\delta$ 7.46-7.41 (m, 4H, H-8), 7.28-7.21 (m, 6H, H-6, H-7), 4.76 (d, $J$ = 5.3 Hz, 1H, H-1), 4.12 (dd, $J$ = 4.7, 3.7 Hz, 1H, H-2), 3.96-3.92 (m, 1H, H-3), 3.71 (ddd, $J$ = 6.4, 5.0, 3.7 Hz, 1H, H-4), 3.62 (dd, $J$ = 11.2, 5.0 Hz, 1H, H-5), 3.54 (dd, $J$ = 11.2, 6.4 Hz, 1H, H-5); $^{13}$C NMR (176 MHz, MeOD) $\delta$ 135.9 (Ar-C), 133.5 (Ar-C), 129.9 (Ar-C), 128.5 (Ar-C), 74.8 (C-2), 73.9 (C-3), 72.4 (C-4), 64.2 (C-5), 63.9 (C-1); $\nu_{\max}$ (solid/cm$^{-1}$) 3384 (O-H), 2919 (O-H), 1084 (C-O); HRMS (ESI) m/z: $[M+H]^+$ Calcd for $C_{17}H_{21}O_4S_2$ 353.0803; found 353.0799.

**(2*R*,3*R*,4*R*)-5,5-bis(phenylthio)pentane-1,2,3,4-tetraol [5c]:[1]**

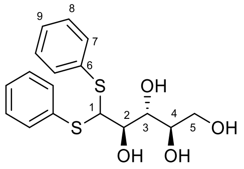


A mixture of D-ribose (13.0 g, 86 mmol) and thiophenol (19.5 mL, 190 mmol, 2.2 eq.) in 90% trifluoroacetic acid in water (21.0 mL) was warmed at 50-60 °C for 12 h whilst stirring. The reaction solution was then concentrated *in vacuo* to a solid residue, which was then purified using silica gel chromatography (EtOAc/petrol 2:3) to yield the product as a yellow oil (6.98 g, 19.8 mmol, 23%): $[\alpha]_D^{21}$= +40 ($c$ = 1.00, MeOH); $^1$H NMR (700 MHz, MeOD) $\delta$ 7.51-7.47 (m, 2H, H-6), 7.34 (d, $J$ = 7.6 Hz, 2H, H-7), 7.28-7.18 (m, 6H, H-8, H-9), 5.02 (d, $J$ = 3.2 Hz, 1H, H-1), 4.41-4.39 (m, H-2), 4.18 (dt, $J$ = 8.4, 4.3 Hz, 1H, H-4), 3.94 (dd, $J$ = 7.9, 7.1 Hz, 1H, H-5), 3.85

(dd, $J$ = 7.8, 7.2 Hz, 1H, H-5), 3.75 (d, $J$ = 8.7 Hz, 1H, H-3); $^{13}$C NMR (176 MHz, MeOD) $\delta$ 136.5 (Ar-C), 132.9 (Ar-C), 130.1 (Ar-C), 128.5 (Ar-C), 78.2 (C-2), 75.8 (C-3), 71.4 (C-4), 65.4 (C-5), 64.1 (C-1); $\nu_{max}$ (film/cm $^{-1}$) 3426 (O-H), 1434 ($CH_2$), 1064 (C-O). Data in accordance with the literature.[1]

**(2*R*,3*R*,4*S*,5*S*)-1,1-bis(phenylthio)hexane-2,3,4,5-tetraol [5d]:[3]**

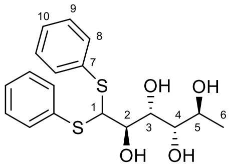


A mixture of L-rhamnose (1.94 g, 10.0 mmol) and thiophenol (2.3 mL, 20.0 mmol, 2.2 eq.) in 90% trifluoroacetic acid in water (5.0 mL) was stirred at 50-60 °C for 12 h. The reaction solution was then concentrated *in vacuo* to a solid residue, which was then recrystallised from boiling EtOAc before washing with $Et_2O$ (2 × 10 mL) and drying *in vacuo* to yield the product as white crystals (2.50 g, 6.00 mmol, 63%): m.p. = 122-126 °C (EtOAc); $[\alpha]_D^{21}$ = +52 ($c$ = 1.00, MeOH); $^1$H NMR (600 MHz, MeOD) $\delta$ 7.54-7.49 (m, 2H, H-8), 7.37-7.32 (m, 2H, H-8), 7.30-7.15 (m, 6H, H-9, H-10), 5.08 (d, $J$ = 1.5 Hz, 1H, H-1), 4.23-4.19 (m, 1H, H-2), 4.12-4.07 (m, 1H, H-3), 3.81-3.74 (m, 1H, H-4), 3.56 (m, 1H, H-5), 1.28-1.24 (m, 3H, H-6); $^{13}$C NMR (176 MHz, MeOD) $\delta$ 136.7 (Ar-C), 132.7 (Ar-C), 129.2 (Ar-C), 128.0 (Ar-C), 74.7 (C-2), 74.1 (C-3), 71.0 (C-4), 69.0 (C-5), 64.7 (C-1), 20.5 (C-6); $\nu_{max}$ (solid/cm $^{-1}$) 3237 (O-H), 1580 (CH), 1064 (C-O); HRMS (ESI) m/z: $[M+Na]^+$ Calcd for $C_{18}H_{22}O_4S_2Na$ 389.0857; found 389.0850.

**(2*R*,3*R*,4*S*,5*S*)-6,6-bis(phenylthio)hexane-1,2,3,4,5-pentaol [5e]:[1]**

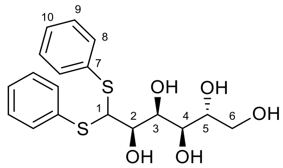


A mixture of D-glucose (1.00 g, 5.60 mmol) and thiophenol (1.3 mL, 12.3 mmol, 2.2 eq.) in 90% trifluoroacetic acid in water (2.00 mL) was stirred at 50-60 °C for 12 h. The reaction solution was then concentrated *in vacuo* to a solid residue, which was then recrystallised from boiling EtOAc before washing with $Et_2O$ (2 × 10 mL) and drying *in vacuo* to yield the product as fine white crystals (12.8 g, 34.0 mmol, 80%): m.p. = 153-156 °C (EtOAc) [158-160 °C][1]; $[\alpha]_D^{21}$ = +44 ($c$ = 1.00, MeOH) [; $^1$H NMR (700 MHz, MeOD) $\delta$ 7.48-7.45 (m, 2H, H-7), 7.44-7.41 (m, 2H, H-8), 7.29-7.21 (m, 6H, H-9, H-10), 4.72 (d, $J$ = 4.9 Hz, 1H, H-1), 4.37-4.33 (m, 1H, H-3), 4.02-3.98 (m, 1H, H-2), 3.78-3.73 (m, 1H, H-4), 3.73-3.67 (m, 1H, H-5), 3.62-3.58 (m, 2H, H-6); $^{13}$C NMR (176 MHz, MeOD) $\delta$ 135.8 (Ar-C), 133.5 (Ar-C), 129.8 (Ar-C), 128.4 (Ar-C), 75.8 (C-2), 73.5 (C-3), 72.9 (C-4), 71.4 (C-5), 64.6 (C-1), 64.1 (C-6); $\nu_{max}$ (solid/cm $^{-1}$) 3286 (O-H), 2931 (O-H), 1438 (CH), 1167 (C-O). Data in accordance with the literature.[1]

**(2*R*,3*S*,4*S*,5*R*)-6,6-bis(phenylthio)hexane-1,2,3,4,5-pentaol [5f]:**[1]

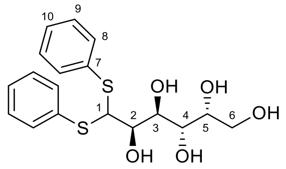

A mixture of D-galactose (1.80 g, 10.0 mmol) and thiophenol (2.26 mL, 22.0 mmol, 2.2 eq.) in 90% trifluoroacetic acid in water (3.0 mL) was stirred at 50-60 °C for 12 h. The reaction solution was then concentrated *in vacuo* to a solid residue, which was then recrystallised from boiling EtOAc before washing with $Et_2O$ (2 × 10 mL) and drying *in vacuo* to yield the product as a white solid (1.93 g, 5.00 mmol, 50%): m.p. = 170-174 °C (EtOAc) [175-176 °C][1]; $[\alpha]_D^{21}$ = −24 (*c* = 1.00, pyridine) [−32, *c* = 1.00, pyridine][1]; $^{1}$H NMR (600 MHz, MeOD) $\delta$ 7.48-7.39 (m, 4H, H-8), 7.31-7.21 (m, 6H, H-9, H-10), 4.78-4.74 (m, 1H, H-1), 4.28 (dd, *J* = 9.1, 1.7 Hz, 1H, H-3), 4.04 (dd, *J* = 8.0, 1.7 Hz, 1H, H-2), 3.93 (td, *J* = 6.3, 1.5 Hz, 1H, H-4) 3.68-3.65 (m, 1H, H-5), 3.64 (d, *J* = 6.3 Hz, 2H, H-6); $^{13}$C NMR (151 MHz, MeOD) $\delta$ 136.4 (Ar-C), 133.9 (Ar-C), 129.8 (Ar-C), 129.0 (Ar-C), 73.1 (C-2), 71.9 (C-3), 71.8 (C-4), 70.8 (C-1), 65.0 (C-5), 64.7 (C-6); $\nu_{max}$ (solid/cm$^{-1}$) 3256 (O-H), 1640 (Ar C=C), 1068 (C-O). Data in accordance with the literature.[1]

**(2*R*,3*R*,4*S*,5*S*)-6-(cyclohexa-2,4-dien-1-ylthio)-6-(phenylthio)hexane-1,2,3,4,5-pentaol [5g]:**[1]

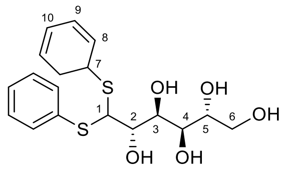

A mixture of D-mannose (3.0 g, 0.017 mmol) and thiophenol (3.84 mL, 0.037 mmol, 2.2 eq.) in 90% trifluoroacetic acid in water (4.30 mL, 3.3 eq) was stirred at 50-60 °C for 12 h. The reaction solution was then concentrated *in vacuo*. Addition of $Et_2O$ (10 mL) resulted in crystallisation of the product as a white solid (3.55 g, 9.30 mmol, 55%): m.p. = 116-119 °C ($Et_2O$) [140-141 °C][1]; $[\alpha]_D^{21}$ = −20 (*c* = 1.00, pyridine) [−30, *c* = 1.2, pyridine][1]; $^{1}$H NMR (700 MHz, MeOD) $\delta$ 7.53-7.49 (m, 2H, H-8), 7.36-7.34 (m, 2H, H-8), 7.29-7.16 (m, 6H, H-9, H-10), 5.07 (d, *J* = 1.4 Hz, 1H, H-1), 4.19 (dd, *J* = 9.4, 0.8 Hz, 1H, H-3), 4.10 (dd, *J* = 9.4, 1.4 Hz, 1H, H-2), 3.81 (dd, *J* = 8.1 Hz, 0.8 Hz, 1H, H-4), 3.79 (dd, *J* = 11.1, 3.6 Hz, 1H, H-6), 3.67-3.64 (m, 1H, H-5), 3.63-3.59 (m, 1H, H-6); $^{13}$C NMR (176 MHz, MeOD) $\delta$ 136.8 (Ar-C), 136.7 (Ar-C), 132.8 (Ar-C), 132.7 (Ar-C), 129.9 (Ar-C), 129.8 (Ar-C), 128.1 (Ar-C), 128.0 (Ar-C), 73.9 (C-2), 73.1 (C-3), 71.2 (C-4), 71.0 (C-5), 65.0 (C-1), 64.8 (C-6); $\nu_{max}$ (solid/cm$^{-1}$) 3355 (O-H), 1428 ($CH_2$), 1067 (C-O); HRMS (ESI) m/z: $[M+Na]^+$ Calcd for $C_{18}H_{22}O_5S_2Na$ 405.0802; found 405.0802. Data in accordance with the literature.[1]

**(2*S*,3*R*)-5,5-bis(Phenylthio)pent-4-ene-1,2,3-triol [6a]:**

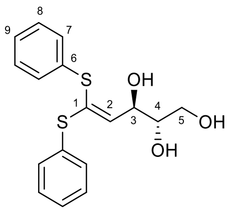

A mixture of **5a** (5.57 g, 15.8 mmol) in MeOH (30.0 mL), with dimethyl carbonate (6.2 mL, 110.6 mmol, 7 eq.) and $K_2CO_3$ (4.37 g, 31.6 mmol, 2 eq.) was stirred at room temperature for 12 h before being filtered. The residue was washed with acetone (2 × 10 mL) and the filtrate concentrated under reduced pressure to yield the product (5.24 g, 15.7 mol, 99%): m.p. = 178-181 °C (MeOH); $[\alpha]_D^{21}$ = +60 ($c$ = 1.00, pyridine); $^1$H NMR (600 MHz, MeOD) $\delta$ 7.33-7.19 (m, 10H, H-7, H-8, H-9), 6.29 (d, $J$ = 8.7 Hz, 1H, H-2), 4.87-4.82 (m, 1H, H-4), 3.70 (m, 2H, H-5), 3.64-3.56 (m, 1H, H-3); $^{13}$C NMR (151 MHz, MeOD) $\delta$ 140.7 (C-1), 133.4 (Ar-C), 132.4 (Ar-C), 130.0 (Ar-C), 129.7 (Ar-C), 128.9 (C-2), 76.2 (C-3), 72.2 (C-4), 64.4 (C-5); $\nu_{\text{max}}$ (solid/cm $^{-1}$) 3265 (O-H), 1610 (Ar C=C), 1022 (C-O); HRMS (ESI) m/z: $[M+H]^+$ Calcd for $C_{17}H_{19}O_3S_2$ 335.0770; found 335.0777.

**(2*R*,3*R*)-5,5-bis(phenylthio)pent-4-ene-1,2,3-triol [6b]:**

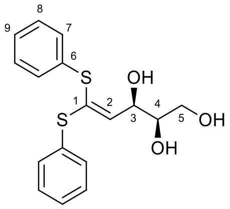

A mixture of **5b** (35 mg, 0.10 mmol) in MeOH (2.0 mL), with dimethyl carbonate (10 µL, 0.15 mmol, 1.5 eq.) and $K_2CO_3$ (28 mg, 0.20 mmol, 2.0 eq.) was stirred at room temperature for 12 h before being concentrated under reduced pressure and purified using silica gel chromatography (acetone/$CH_2Cl_2$ 2:3) to yield the product as a yellow oil (160 mg, 0.05 mmol, 48%); $[\alpha]_D^{21}$= −44 ($c$ = 1.00, MeOH); $^1$H NMR (700 MHz, MeOD) $\delta$ 7.31-7.23 (m, 10H, H-7, H-8, H-9), 6.23 (d, $J$ = 8.8 Hz, 1H, H-2), 4.80 (dd, $J$ = 8.7, 4.6 Hz, 1H, H-3), 3.62 (dd, $J$ = 10.9, 6.9 Hz, 1H, H-5), 3.59-3.55 (m, 1H, H-4), 3.50 (dd, $J$ = 10.9, 6.7 Hz, 1H, H-5); $^{13}$C NMR (176 MHz, MeOD) $\delta$ 140.3 (C-1), 134.7 (Ar-C), 134.5 (Ar-C), 134.3 (Ar-C), 133.5 (Ar-C), 132.2 (Ar-C), 130.0 (Ar-C), 129.7 (Ar-C), 129.0 (Ar-C), 128.3 (C-2), 76.1 (C-3), 71.6 (C-4), 64.2 (C-5); $\nu_{\text{max}}$ (film/cm $^{-1}$) 3329 (O-H), 1640 (Ar C=C), 1580 (C=C), 1474 (CH), 1023 (C-O); HRMS (ESI) m/z: $[M+H]^+$ Calcd for $C_{17}H_{19}O_3S_2$ 335.0770; found 335.0776.

**(2*S*,3*S*)-5,5-bis(phenylthio)pent-4-ene-1,2,3-triol [*ent*-6b]:**

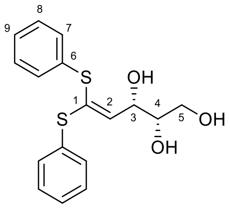

A mixture of ***ent*-5b** (300 mg, 0.85 mmol) in MeOH (2.00 mL), with dimethyl carbonate (71 μL, 1.28 mmol, 1.5 eq.) and $K_2CO_3$ (235 mg, 1.7 mmol, 2.0 eq) was stirred at room temperature for 12 h before being filtered and washed with MeOH (2 × 5 mL). The mixture was then concentrated under reduced pressure to yield the product (224 mg, 0.67 mmol, 79%): m.p. = 196-198 °C (MeOH), $[\alpha]_D^{21}$= +64 (*c* = 1.00, pyridine); $^1$H NMR (700 MHz, MeOD) $\delta$ 7.32-7.23 (m, 10H, H-7, H-8, H-9), 6.23 (d, *J* = 8.8 Hz, 1H, H-2), 4.79 (dd, *J* = 7.7, 3.8 Hz, 1H, H-3), 3.62 (dd, *J* = 10.9, 4.6 Hz, 1H, H-5), 3.59-3.56 (m, 1H, H-4), 3.52-3.48,(dd, *J* = 10.9, 4.7 Hz 1H, H-5); $^{13}$C NMR (176 MHz, MeOD) $\delta$ 140.3 (C-1), 134.7 (Ar-C), 134.5 (Ar-C), 134.3 (Ar-C), 133.5 (Ar-C), 132.2 (Ar-C), 130.0 (Ar-C), 129.7 (Ar-C), 129.0 (Ar-C), 128.2 (C-2), 76.2 (C-3), 71.6 (C-4), 64.2 (C-4); $v_{\max}$ (solid/cm $^{-1}$) 3364 (O-H), 1580 (C=C), 1475 (CH), 1068 (C-O); HRMS (ESI) m/z: $[M+H]^+$ Calcd for $C_{17}H_{19}O_3S_2$ 335.0770; found 335.0777.

**(2*R*,3*S*,4*R*)-6,6-bis(phenylthio)hex-5-ene-1,2,3,4-tetraol [6e]:**

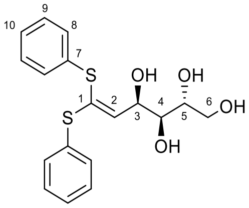

A mixture of **5e** (1.3 g, 3.4 mmol) in MeOH (20 mL), with dimethyl carbonate (285 μL, 5.1 mmol, 1.5 eq.) and $K_2CO_3$ (940 mg, 6.8 mmol, 2.0 eq) was stirred at room temperature for 12 h. The mixture was concentrated under reduced pressure and the residue washed with water to yield the product as a yellow solid (1.18 g, 3.24 mmol, 95%): m.p. = 144-148 °C (EtOAc), $[\alpha]_D^{25}$= −32 (*c* = 1.00, pyridine); $^1$H NMR (700 MHz, MeOD) $\delta$ 7.30-7.20 (m, 10H, H-8, H-9, H-10), 6.42 (d, *J* = 8.6 Hz, 1H, H-2), 5.07 (d, *J* = 9.7 Hz, 1H, H-3), 3.80-3.75 (dd, *J* = 11.0, 3.3 Hz, 1H, H-6), 3.73-3.68 (m, 1H, H-5), 3.66-3.62 (m, 1H, H-4), 3.47 (dd, *J* = 7.7, 2.7 Hz, 1H, H-6); $^{13}$C NMR (176 MHz, MeOD) $\delta$ 141.4 (C-1), 133.6 (Ar-C), 133.3 (Ar-C), 132.2 (Ar-C), 130.0 (Ar-C), 129.7 (C-2), 72.5 (C-3), 69.4 (C-4), 63.3 (C-5), 62.4 (C-6); $v_{\max}$ (film/cm$^{-1}$) 3253 (O-H), 1641 (Ar C=C), 1013 (C-O); HRMS (ESI) m/z: $[M+H]^+$ Calcd for $C_{18}H_{21}O_4S_2$ 365.0876; found 365.0898.

**(2*R*,3*R*,4*R*)-6,6-bis(phenylthio)hex-5-ene-1,2,3,4-tetraol [6f]:**

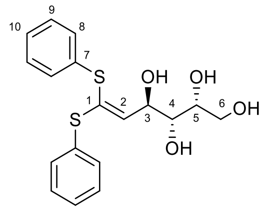


A mixture of **5f** (640 mg, 1.67 mmol) in MeOH (2.6 mL) with dimethyl carbonate (141 µL, 2.51 mmol, 1.5 eq.) and $K_2CO_3$ (463 mg, 3.35 mmol, 2.0 eq) was stirred at room temperature for 12 h. The mixture was concentrated under reduced pressure and purified using silica gel chromatography (acetone/$CH_2Cl_2$, 1:5) to yield the product (322 mg, 0.88 mmol, 53%): m.p. = 80-84 °C ($CH_2Cl_2$), $[\alpha]_D^{21}$= +28 (*c* = 1.00, pyridine); $^1$H NMR (600 MHz, MeOD) $\delta$ 7.34-7.17 (m, 10H, H-8, H-9, H-10), 6.29 (d, *J* = 8.7 Hz, 1H, H-2), 4.90 (dd, *J* = 8.7, 7.2 Hz, 1H, H-3), 3.88-3.84 (m, 1H, H-5), 3.67-3.58 (m, 2H, H-6), 3.55 (dd, *J* = 7.2, 3.5 Hz, 1H, H-4); $^{13}$C NMR (151 MHz, MeOD) $\delta$ 141.9 (C-1), 135.0 (Ar-C), 135.0 (Ar-C), 134.8 (Ar-C), 133.2 (Ar-C), 132.4 (Ar-C), 130.0 (Ar-C), 129.7 (Ar-C), 128.8 (Ar-C), 128.3 (C-2), 74.4 (C-3), 72.2 (C-4), 71.5 (C-5), 64.7 (C-6); $\nu_{\max}$ (solid/cm $^{-1}$) 3336 (O-H), 1578 (C=C), 1438 ($CH_2$); HRMS (ESI) m/z: $[M+H]^+$ Calcd for $C_{18}H_{21}O_4S_2$ 365.0676; found 365.0668.

**(2*R*,3*R*,4*R*)-5,5-bis(phenylthio)pentane-1,2,3,4-tetrayl tetraacetate [pre-8c]:**

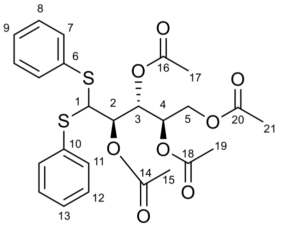


A mixture of **5c** (1.27 g, 3.56 mmol) in dry pyridine (10 mL) and acetic anhydride (1.7 µL, 18.0 mmol, 5 eq.) was kept overnight at room temperature with stirring. The mixture was poured onto crushed ice and extracted with $CH_2Cl_2$ (10 mL). The organic layer was washed with *aq.* 15% $CuSO_4$ solution (10 mL) followed by brine (10 mL) before drying over $MgSO_4$. The mixture was filtered through silica and concentrated *in vacuo* to give the product as a colourless oil (1.81 g, 3.5 mmol, 98%): $[\alpha]_D^{21}$= +52 (*c* = 1.00, $CHCl_3$); $^1$H NMR (700 MHz $CDCl_3$) $\delta$ 7.45-7.43 (m, 2H, H-11), 7.36-7.32 (m, 2H, H-7), 7.28-7.19 (m, 6H, H-8, H-9, H-12, H-13), 5.66 (dd, *J* = 7.3, 3.8 Hz, 1H, H-2), 5.41 (dd, *J* = 7.3, 3.5 Hz, 1H, H-3), 5.27 (m, *J* = 7.4, 3.6 Hz, 1H, H-4), 4.44 (d, *J* = 3.6 Hz, 1H, H-1), 4.23 (dd, *J* = 12.1, 3.4 Hz, 1H, H-5), 4.04 (dd, *J* = 12.1, 7.4 Hz, 1H, H-5), 2.07 (s, 3H, $CH_3$), 1.95 (s, 3H, $CH_3$), 1.94-1.91 (s, 3H, $CH_3$), 1.91 (s, 3H, $CH_3$); $^{13}$C NMR (176 MHz, $CDCl_3$) $\delta$ 170.5 (C=O), 169.9 (C=O), 169.7 (C=O), 169.2 (C=O), 133.8 (Ar-C), 132.6 (Ar-C), 128.6 (Ar-C), 128.3 (Ar-C), 72.38 (C-1), 70.6 (C-2), 70.1 (C-3), 61.8 (C-4), 61.3 (C-5), 20.8 ($CH_3$), 20.7 ($CH_3$), 20.7 ($CH_3$), 20.6 ($CH_3$); $\nu_{\max}$ (film/cm $^{-1}$) 1739 (C=O), 1643 (Ar C=C), 1367 (CH), 1197 (C-O); HRMS (ESI) m/z: $[M+H]^+$ Calcd for $C_{25}H_{29}O_8S_2$ 521.1226; 521.1336 found.

**(2*R*,3*S*)-5,5-bis(phenylthio)pent-4-ene-1,2,3-triol [8c]:**

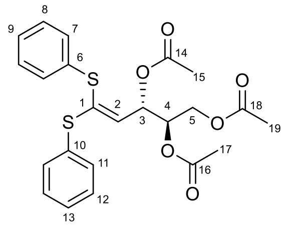

A mixture of **pre-8c** (1.8 g, 3.5 mmol) dissolved in DMSO-$d_6$ (10 mL) and DBU (1.1 mL, 7.0 mmol, 2.0 eq) was stirred at room temperature for 12 h to yield the ketene thioacetal. The mixture was added to water (10 mL) and the product extracted with $Et_2O$ (5 x 10 mL), dried over $MgSO_4$ and concentrated *in vacuo* to yield the isolated product as a yellow oil (1.55 g, 3.4 mmol, 96%): $[\alpha]_D^{21}$= +96 (*c* = 1.00, $CHCl_3$); $^1$H NMR (700 MHz, $CDCl_3$) $\delta$ 7.36-7.21 (m, 10H, H-6, H-7, H-8, H-9, H-10, H-11, H-12, H-13), 6.11 (dd, *J* = 8.8, 5.7 Hz, 1H, H-2), 5.83 (d, *J* = 8.8 Hz, 1H, H-4), 5.28 (ddd, *J* = 6.5, 5.8, 3.8 Hz, 1H, H-3), 4.26 (dd, *J* = 12.0, 3.7 Hz, 1H, H-5), 4.17 (dd, *J* = 12.0, 6.3 Hz, 1H, H-5), 2.07 (s, 3H, $CH_3$), 2.051 (s, 3H, $CH_3$), 2.049 (s, 3H, $CH_3$); $^{13}$C NMR (176 MHz, $CDCl_3$) $\delta$ 170.7 (C=O), 170.0 (C=O), 169.6 (C=O), 140.4 (C-1), 133.4 (Ar-C), 132.7 (Ar-C), 132.5 (Ar-C), 132.4 (Ar-C), 129.5 (Ar-C), 129.2 (Ar-C), 128.9 (Ar-C), 128.5 (Ar-C), 128.0 (C-2), 71.3 (C-3), 70.8 (C-4), 62.2 (C-5), 21.0 ($CH_3$), 21.0 ($CH_3$), 20.9 ($CH_3$); $\nu_{max}$ (film/cm $^{-1}$) 1745 (C=O), 1672 (C=C), 1370 (CH), 1024 (C-O); HRMS (ESI) m/z: $[M+H]^+$ Calcd for $C_{23}H_{25}O_6S_2$ 461.1048; 461.1113 found.

**(2*R*,3*R*,4*S*,5*S*)-1,1-bis(phenylthio)hexane-2,3,4,5-tetrayl tetraacetate [pre-8d]:**

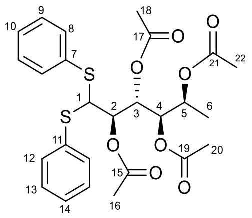

A mixture of **5d** (500 mg, 1.26 mmol) in anhydrous pyridine (5 mL) and acetic anhydride (596 µL, 6.30 mmol, 5 eq.) was stirred at room temperature for 4 h. An additional aliquot of acetic anhydride (596 µL, 6.30 mmol, 5 eq.) was added and the mixture stirred for a further 4 h. The resulting mixture was poured onto crushed ice and extracted with $CH_2Cl_2$ (10 mL). The organic layer was washed with *aq.* 15% $CuSO_4$ solution (10 mL) followed by brine (10 mL) before drying over $MgSO_4$. The mixture was filtered through silica and concentrated *in vacuo*. The mixture was purified via column chromatography (3:2 EtOAc/Petrol) to give the pure product as a yellow oil (630 mg, 1.18 mmol, 94%): $[\alpha]_D^{25}$= +92 (*c* = 1.00, $CHCl_3$); $^1$H NMR (700 MHz, $CDCl_3$) $\delta$ 7.58-7.56 (m, 2H, H-8), 7.36-7.30 (m, 5H, H-12, H-13, H-14), 7.26 (m, 3H, H-9, H-10), 5.88 (dd, *J* = 8.6, 1.8 Hz, 1H, H-3), 5.33 (dd, *J* = 8.6, 2.9 Hz, 1H, H-2), 5.20 (dd, *J* = 8.3, 1.8 Hz, 1H, H-4), 4.88-4.81 (m, 1H, H-5), 4.38 (d, *J* = 2.9 Hz, 1H, H-1), 2.06 (s, 3H, $CH_3$), 2.01 (s, 3H, $CH_3$), 1.98 (s, 6H, $CH_3$ x 2), 1.16 (d, *J* = 6.4 Hz, 3H, H-6); $^{13}$C NMR (176 MHz, $CDCl_3$) $\delta$ 170.4 (C=O), 170.2 (C=O), 169.8 (C=O), 169.6 (C=O), 134.2 (Ar-C), 133.9 (Ar-C), 133.5 (Ar-C), 129.1 (Ar-C), 128.4 (Ar-C), 71.5 (C-2), 71.4 (C-3), 69.0 (C-4), 67.3 (C-5), 61.6 (C-1), 21.3

(CH3), 20.9 (CH3), 20.8 (CH3), 20.8 (CH3), 16.6 (C-6); $v_{\text{max}}$ (film/cm $^{-1}$) 1730 (C=O), 1371 (CH3), 1235 (C-O), 1043 (C-O); HRMS (ESI) m/z: [M +Na]$^+$ Calcd for $C_{26}H_{30}O_8S_2Na$ 557.1274; found 557.1274.

**(2*S*,3*R*,4*S*)-6,6-bis(Phenylthio)hex-5-ene-2,3,4-triyl triacetate [8d]:**

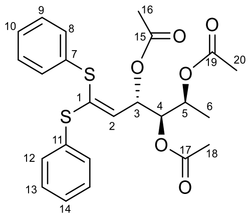

A mixture of **pre-8d** (95 mg, 0.17 mmol) and triazabicyclodecene (37 mg, 2mmol, 2.0 eq) in DMSO-$d_6$ (1.2 mL) was stirred at room temperature for 18 h. The mixture was added to water (10 mL) and the product extracted with $Et_2O$ (5 x 10 mL), dried over $MgSO_4$ and concentrated *in vacuo.* The mixture was purified via column chromatography (2:3 EtOAc/Petrol) to give the pure product as a yellow oil (62 mg, 0.13 mmol, 74%): $[\alpha]_D^{25}$= +96 ($c$ = 0.80, $CHCl_3$); $^1$H NMR (700 MHz, $CDCl_3$) $\delta$ 7.36-7.28 (m, 10H, H-8, H-9, H-10), 6.17 (dd, $J$ = 8.7, 5.0 Hz, 1H, H-3), 5.69 (d, $J$ = 8.7 Hz, 1H, H-2), 5.17 (dd, $J$ = 6.1, 5.0 Hz, 1H, H-4), 5.04-5.02 (m, 1H, H-5), 2.07 (s, 3H, CH3), 2.04 (s, 3H, CH3), 2.02 (s, 3H, CH3), 1.23 (d, $J$ = 6.4 Hz, 3H, H-6); $^{13}$C NMR (176 MHz, $CDCl_3$) $\delta$ 170.2 (C=O), 170.0 (C=O), 169.7 (C=O), 133.8 (Ar-C), 132.2 (Ar-C), 129.3 (Ar-C), 129.0 (Ar-C), 128.6 (C-1), 127.9 (C-2), 74.7 (C-3), 70.3 (C-4), 67.9 (C-5), 21.2 (CH3), 21.1 (CH3), 20.8 (CH3), 16.0 (C-6); $v_{\text{max}}$ (film/cm $^{-1}$) 1746 (C=O), 1653 (C=C), 1370 (CH3), 1217 (C-O), 1056 (C-O); HRMS (ESI) m/z: [M+H]$^+$ Calcd for $C_{24}H_{27}O_6S_2$ 475.1244; found 475.1250.

**(2*R*,3*R*,4*S*,5*S*)-6,6-bis(phenylthio)hexane-1,2,3,4,5-pentayl pentaacetate [pre-8g]:**

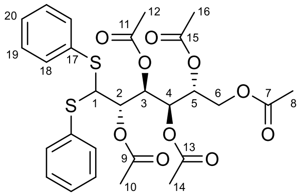

A mixture of **5g** (293 mg, 0.766 mmol) in anhydrous pyridine (3 mL) and acetic anhydride (1.74 mL, 18.40 mmol, 24 eq.) was added over 4 h at room temperature. The resultant mixture was stirred overnight before it was poured onto crushed ice and extracted with $CH_2Cl_2$ (10 mL). The organic layer was washed with *aq.* 15% $CuSO_4$ solution (10 mL) followed by brine (10 mL) before drying over $MgSO_4$. The mixture was filtered through silica and concentrated *in vacuo* to give pure product as a colourless oil (421 mg, 0.765 mmol, 99%): $[\alpha]_D^{25}$= +32 ($c$ = 1.00, $CHCl_3$); $^1$H NMR (700 MHz, MeOD) $\delta$ 7.58-7.53 (m, 4H, H-18), 7.38-7.23 (m, 6H, H-19, H-20), 5.84 (dd, $J$ = 8.3, 1.3 Hz, 1H, H-3), 5.44 (dd, $J$ = 9.1, 1.2 Hz, 1H, H-4), 5.33 (dd, $J$ = 8.2, 3.5 Hz, 1H, H-2), 5.05-4.99 (m, 1H, H-5), 4.42 (d, $J$ = 3.5 Hz, 1H, H-1), 4.19 (dd, $J$ = 12.5, 2.6 Hz, 1H, H-6), 4.03 (dd, $J$ = 12.5, 5.1 Hz, 1H, H-6), 2.10 (s, 3H, CH3), 2.03 (s, 3H, CH3), 2.00 (s, 3H, CH3), 1.99 (s, 3H, CH3), 1.96 (s, 3H, CH3); $^{13}$C NMR (176 MHz, MeOD) $\delta$ 170.7 (C=O), 170.1 (C=O), 170.1 (C=O), 169.74 (C=O × 2), 134.0 (Ar-C), 133.8 (Ar-C), 133.5 (Ar-C), 133.4 (Ar-C), 129.2 (Ar-C), 129.1 (Ar-C), 128.5 (Ar-C), 128.4 (Ar-C), 71.5 (C-3), 69.0

(C-5), 68.1 (C-2), 67.7 (C-4), 62.0 (C-6), 61.3 (C-1), 21.1 ($CH_3$), 20.9 ($CH_3$), 20.8 ($CH_3$), 20.8 ($CH_3$), 20.7 ($CH_3$); $\nu_{max}$ (film/cm$^{-1}$) 1744 (C=O), 1368 ($CH_2$), 1209 (C-O), 1043 (C-O); HRMS (ESI) m/z: [M +Na]$^+$ Calcd for $C_{28}H_{32}O_{10}S_2Na$ 573.1223; found 573.1223.

**(2*R*,3*S*,4*R*)-6,6-bis(Phenylthio)hex-5-ene-1,2,3,4-tetrayl tetraacetate [8g]:**

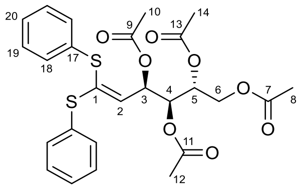

A mixture of **pre-8g** (21 mg, 0.0382 mmol) and $^t$BuOK (10 mg, 0.0870 mmol, 4.4 eq) was dissolved in DMSO-$d_6$ (1 mL) and stirred at room temperature for 12 h to yield the ketene thioacetal. The mixture was added to water (5 mL) and the product extracted with $Et_2O$ (4 × 5 mL), dried over $MgSO_4$ and concentrated *in vacuo* to yield the product as an inseperable mixture of the product and starting material as a yellow oil (4 mg, 0.018 mmol, 47%). Yield calculated by $^1$H NMR via addition of an internal standard (1,4-Dimethoxybenzene). The product was characterised by preparation of an authentic sample via acetylation of compound **6e** (see below).

A mixture of **6e** (100 mg, 0.275 mmol) in anhydrous pyridine (1 mL) and acetic anhydride (130 μL, 1.37 mmol, 6 eq.) was left to stir for 4 h at room temperature, with addition of acetic anhydride (130 μL, 1.37 mmol, 6 eq.) repeated every hour for 4 h. The resultant mixture was stirred overnight before it was poured onto crushed ice and mixed with $CH_2Cl_2$ (10 mL). The organic layer was washed with *aq.* 15% $CuSO_4$ solution (10 mL) followed by brine (10 mL) before drying over $MgSO_4$. The mixture was filtered through silica and concentrated *in vacuo* to give the product as a colourless oil (64 mg, 0.12 mmol, 44%): $[\alpha]_D^{25}$ = −20 (*c* = 1.00, $CHCl_3$); $^1$H NMR (700 MHz, $CDCl_3$) $\delta$ 7.38-7.35 (m, 2H, H-20), 7.33-7.26 (m, 8H, H-18, H-19), 6.20 (dd, *J* = 8.5, 3.5 Hz, 1H, H-3), 5.62 (d, *J* = 8.5 Hz, 1H, H-2), 5.30 (dd, *J* = 8.0, 3.6 Hz, 1H, H-4), 5.21 (m, 1H, H-5), 4.22 (dd, *J* = 12.4, 2.6 Hz, 1H, H-6), 4.16 (dd, *J* = 12.4, 5.4 Hz, 1H, H-6), 2.06 (s, 1H, $CH_3$), 2.05 (s, 1H, $CH_3$), 2.04 (s, 1H, $CH_3$), 2.04 (s, 1H, $CH_3$); $^{13}$C NMR (176 MHz, $CDCl_3$) $\delta$ 170.7 (C=O), 170.0 (C=O), 169.7 (C=O), 169.5 (C=O), 139.1 (C-1), 133.6 (C-2), 132.8 (Ar-C), 132.2 (Ar-C), 132.0 (Ar-C), 129.3 (Ar-C), 129.0 (Ar-C), 128.7 (Ar-C), 128.6 (Ar-C), 127.9 (Ar-C), 71.3 (C-4), 70.0 (C-5), 68.7 (C-3), 62.1 (C-6), 21.0 ($CH_3$), 20.9 ($CH_3$), 20.8 ($CH_3$), 20.7 ($CH_3$); $\nu_{max}$ (film/cm$^{-1}$) 1744 (C=O), 1660 (C=C), 1370 ($CH_2$), 1210 (C-O), 1024 (C-O), 774 (C=C); HRMS (ESI) m/z: [M+H]$^+$ Calcd for $C_{26}H_{29}O_8S_2$ 533.1298; found 533.1298.

**(2*S*, 3*R*)-2,3-Dihydroxypentyl benzoate [10]:**

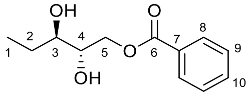

Adapted from a literature procedure.[5,6] A solution of **6a** (67 mg, 0.2 mmol) in EtOH (2 mL) with Raney-Ni (2 g) was stirred at RT for 12 h. The mixture was filtered through a plug of

silica and washed with EtOH (5 mL). The filtrate was concentrated *in vacuo* and dissolved in pyridine (3 mL) at 0 °C under an inert atmosphere. To the stirred solution was added benzoyl chloride (23 µL, 0.2 mmol) dropwise. The resultant mixture was stirred at 0 °C for 2 h and then at RT for 12 h. The solvent was evaporated and then co-evaporated with toluene (5 mL). The residue was slowly poured into vigorously stirring water. The product was extracted with EtOAc (3 × 5 mL) before washing with brine (5 mL) and sat. *aq.* $NaHCO_3$ (5 mL). The mixture was dried using $MgSO_4$ and concentrated *in vacuo*. The crude product was purified using column chromatography (20% EtOAc/petrol) to yield the product as a colourless oil (42 mg, 0.19 mmol, 94%): $[\alpha]_D^{25}$= +4 (*c* = 1.00, pyridine); $^1$H NMR (700 MHz, $CDCl_3$) $\delta$ 8.06 (d, *J* = 7.8 Hz, 2H, H-8), 7.59 (t, *J* = 7.3 Hz, 1H, H-10), 7.46 (t, *J* = 7.5 Hz, 2H, H-9), 4.52 (d, *J* = 4.8 Hz, 2H, H-5), 3.94-3.88 (m, 1H, H-4), 3.67 (m, 1H, H-3), 2.54 (d, *J* = 4.7 Hz, 1H, OH), 2.15 (d, *J* = 4.5 Hz, 1H, OH), 1.72-1.65 (m, 1H, H-2), 1.61-1.56 (m, 1H, H-2), 1.04 (t, *J* = 7.4 Hz, 3H, H-1); $^{13}$C NMR (176 MHz, $CDCl_3$) $\delta$ 167.4 (C-6), 133.5 (Ar-C), 130.0 (Ar-C), 129.8 (Ar-C), 128.6 (Ar-C), 74.0 (C-5), 73.1 (C-4), 66.3 (C-3), 25.6 (C-2), 10.3 (C-1); $\nu_{max}$ (film/cm $^{-1}$) 3386 (O-H), 1680 (C=O), 1582 ($CH_2$), 1067 (C-O); HRMS (ESI) m/z: $[M+Na]^+$ Calcd for $C_{12}H_{16}O_4Na$ 247.0491; found 247.0491.

**Methyl-(2*R*,3*R*,4*R*,5*S*)-4-Hydroxy-5-(hydroxymethyl)-2-phenyltetrahydrofuran-3-carboxylate [13]:**

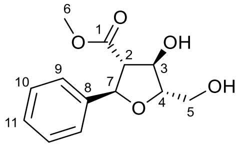


Adapted from a literature procedure.[7] A mixture of **6a** (50 mg, 0.15 mmol) in $CH_2Cl_2$ under Ar was stirred at −78 °C for 5 min before the addition of benzaldehyde dimethylacetal (24 µL, 0.16 mmol) and borane trifluoride-tetrahydrofuran complex (18 µL, 0.16 mmol). The solution was left to stir for 1 h. The solution was concentrated *in vacuo*, followed by addition of EtOAc (5 mL). The product was extracted with EtOAc (3 × 10 mL), washed over brine (10 mL) and dried over $MgSO_4$. The product was purifed via silica chromatography in a gradient of EtOAc/Petrol and isolated as a yellow oil (12 mg, 0.04 mmol, 25%): $[\alpha]_D^{25}$= −60 (*c* = 1.00, pyridine); $^1$H NMR (700 MHz, $CDCl_3$) $\delta$ 7.34-7.28 (m, 5H, H-9, H-10, H-11), 5.14 (d, *J* = 8.9 Hz, 1H, H-7), 4.21-4.18 (m, 1H, H-4), 4.07-4.01 (m, 1H, H-3), 3.88 (dd, *J* = 12.0, 5.3 Hz, 1H, H-5), 3.79-3.72 (m, 1H, H-5), 3.56 (s, 3H, H-6), 3.12 (dd, *J* = 10.0, 8.9 Hz, 1H, H-2); $^{13}$C NMR (176 MHz, $CDCl_3$) $\delta$ 171.6 (C-1), 134.0 (Ar-C), 129.3 (Ar-C), 128.8 (Ar-C), 125.9 (Ar-C), 84.7 (C-4), 83.4 (C-7), 62.4 (C-3), 60.2 (C-5), 52.4 (C-6), 50.9 (C-2); $\nu_{max}$ (film/cm $^{-1}$) 2919 (O-H), 1734 (C=O), 1461 ($CH_2$); HRMS (ESI) m/z: $[M]^-$ Calcd for $C_{13}H_{15}O_5$ 251.0697; found 251.0875.

**(4*S*, 5*S*)-5-(Hydroxymethyl)-4-(phenylthio)dihydrofuran-2(3H)-one and (4*R*, 5*S*)-5-(hydroxymethyl)-4-(phenylthio)dihydrofuran-2(3H)-one [14]:**

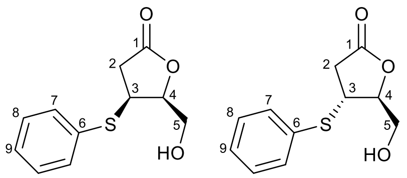

A mixture of **6a** (100 mg, 0.33 mmol) and In(OTf)$_3$ (675 mg, 1.2 mmol, 4 eq.) were combined and stirred neat at room temperature for 4 h. The crude mixture was purified using column chromatography (2:1 EtOAc/Petrol) to yield the isolated lactone as an oil, a mixture of two isomers (2:1) (39 mg, 0.174 mmol, 53%). (4*R*): $^1$H NMR (700 MHz, CDCl$_3$) $\delta$ 4.45-4.41 (m, 1H, H-4), 3.98-3.91 (m, 2H, H-5, H-3), 3.69-3.63 (dd, $J$ = 13.3, 3.5 Hz, 1H, H-5), 3.08-2.99 (dd, $J$ = 17.5, 8.5 Hz, 1H, H-2), 2.63-2.56 (dd, $J$ = 17.5, 8.4 Hz, 1H, H-2); $^{13}$C NMR (176 MHz, CDCl$_3$) $\delta$ 174.56 (C-1) 133.1 (Ar-C), 131.4 (Ar-C), 129.6 (Ar-C), 128.7 (Ar-C), 85.0 (C-4), 62.5 (C-2), 42.2 (C-3), 36.4 (C-5). (4*S*): $^1$H NMR (700 MHz, CDCl$_3$) $\delta$ 4.77-4.74 (m, 1H, H-4), 4.19-4.13 (m, 2H, H-59), 4.11 (dd, $J$ = 12.6, 4.5 Hz, 1H, H-3), 4.00-3.96 (m, 1H, H-4), 2.90 (dd, $J$ = 17.6, 8.7 Hz, 1H, H-2), 2.76 (dd, $J$ = 17.6, 8.6 Hz, 1H, H-2); $^{13}$C NMR (176 MHz, CDCl$_3$) $\delta$ 174.4 (C-1), 131.5 (Ar-C), 129.7 (Ar-C), 128.7 (Ar-C), 128.1 (Ar-C), 81.8 (C-4), 62.5 (C-2), 44.6 (C-3), 29.8 (C-5); $\nu_{\max}$ (film/cm$^{-1}$) 3390 (O-H), 1781 (C=O), 1651 (Ar-CH), 1176 (CH$_2$); HRMS (ESI) m/z: $[M+H]^+$ Calcd for $C_{11}H_{13}O_3S$ 225.0580; found 225.0580. Data in accordance with the literature.[8]

# DFT Calculations

Conformational searches on tetraols *ent*-**5a** and **5c**, and on cyclic carbonates *ent*-**7a** and **7c**, were carried out using the MMFF force field and the SCAN function in Tinker.[9] All structures within 10.0 kJ mol$^{-1}$ of the lowest-energy structure for each compound were then optimised in the gas phase using Gaussian 09,[10] with the M06-2X functional and 6-31G(d,p) basis set. All structures were confirmed as minima by the absence of imaginary vibrational frequencies. Duplicate structures were removed, then solvation energies for each of the unique minima was determined by a single-point solution-phase energy calculation (IEFPCM, methanol). Free energies were corrected for low-frequency vibrations using *GoodVibes*.[11]

The lowest-energy conformation of each compound is depicted overleaf.[12] Coordinates are provided in xyz format in an accompanying zip file.

| Compound | $E_{gas}$/Hartree | $G_{gas}$/Hartree | $E_{MeOH}$/Hartree | $G_{MeOH}$/Hartree[a] | $G_{rel}$/kJ mol$^{-1}$ [b] |
|---|---|---|---|---|---|
| *ent*-**5a** | −1756.661759 | −1756.366811 | −1756.676496 | −1756.381547 | 12.3 |
| **5b** | −1756.670599 | −1756.373674 | −1756.683212 | −1756.386287 | 0.0 |
| *ent*-**7a** | −1868.772929 | −1868.492095 | −1868.791090 | −1868.510257 | 0.0 |
| **7b** | −1868.768289 | −1868.487270 | −1868.788221 | −1868.507202 | 9.0 |

All calculations at the M06-2X/6-31G(d,p) level.

[a]Calculated as $G_{MeOH} = E_{MeOH} + (G_{gas} - E_{gas})$.

[b]Free energy relative to the lower of the two stereoisomers considered.

| | |
|---|---|
| 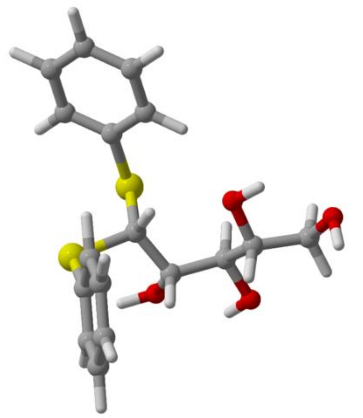<br>*ent*-**5a** | 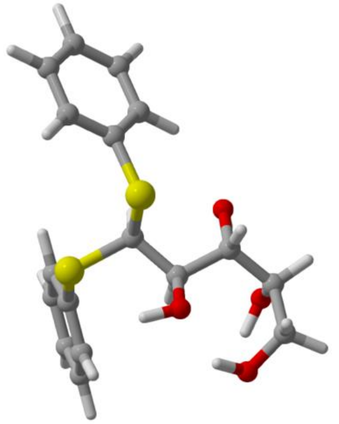<br>**5c** |
| 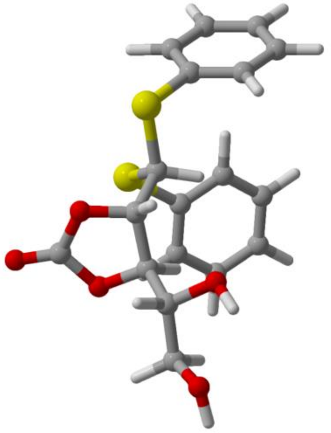<br>*ent*-**7a** | 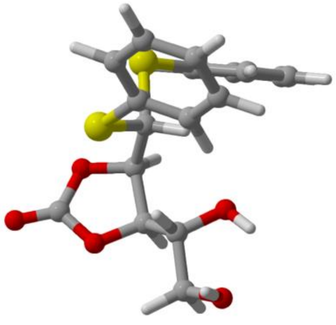<br>**7c** |

# References


(1) Funabashi, M.; Arai, S.; Shinohara, M. Novel Syntheses of Diphenyl and/or Trimethylene Dithioacetals of Mono- and Oligosaccharides in 90% Trifluoroacetic Acid. *J. Carbohydr. Chem.* **1999**, *18*, 333–341.

(2) Horton; Wander, Diphenyl dithioacetals of D-ribose, D-xylose, and D- and L-arabinose. Conformational studies and formation of a ketene diphenyldithioacetal. *Carbohydrate Research*, **1970**, *13*, 33-45.

(3) Fragoso-Serrano, M.; Guillén-Jaramillo, G.; Pereda-Miranda, R.; M. Cerda-García-Rojas, C. Conformational Analysis of Sulfur-Containing 6-Deoxy-l-Hexose Derivatives by Molecular Modeling and NMR Spectroscopy. A Theoretical Study and Experimental Evidence of Intramolecular Nonbonded Interactions between Sulfur and Oxygen. *J. Org. Chem.* **2003**, *68*, 7167–7175.

(4) Pereda-Miranda, R.; Fragoso-Serrano, M.; Cerda-García-Rojas, C. M. Application of Molecular Mechanics in the Total Stereochemical Elucidation of Spicigerolide, a Cytotoxic 6-Tetraacetyl-Oxyheptenyl-5,6-Dihydro-α-Pyrone from Hyptis Spicigera. *Tetrahedron* **2001**, *57*, 47–53.

(5) Tchertchian, S.; Vallée, Y. Stereoselective Synthesis of Hydroxy-Ketenedithioacetals from Aldehydes. *Tetrahedron* **1998**, *54*, 7777–7786.

(6) Bourne, E. J; Lees, E.M; Weigel, H. Phenylboronates of Acyclic Polyhydroxy-compounds. *J. Chem. Soc.* **1964**, 3798–3802.

(7) Saitoh, T.; Jimbo, N.; Ichikawa, J. A Novel Synthesis of Syn and Anti β-Hydroxy Dithioacetals, Masked Cross-Aldols between Aldehydes. *Chem. Lett.* **2004**, *33*, 1032–1033.

(8) Takano, S.; Kurotaki, A.; Takahashi, M.; Ogasawara, K. Practical Synthesis of Some Versatile Chiral Building Blocks from (D)-Mannitol. *Synthesis* **1986**, 403-406.

(9) Ponder, J. W., *Tinker 7.1*, **2015**, http://dasher.wustl.edu/tinker.

(10) *Gaussian 09, Revision D.01,* Frisch, M. J.; Trucks, G. W.; Schlegel, H. B.; Scuseria, G. E.; Robb, M. A.; Cheeseman, J. R.; Scalmani, G.; Barone, V.; Mennucci, B.; Petersson, G. A.; Nakatsuji, H.; Caricato, M.; Li, X.; Hratchian, H. P.; Izmaylov, A. F.; Bloino, J.; Zheng, G.; Sonnenberg, J. L.; Hada, M.; Ehara, M.; Toyota, K.; Fukuda, R.; Hasegawa, J.; Ishida, M.; Nakajima, T.; Honda, Y.; Kitao, O.; Nakai, H.; Vreven, T.; Montgomery, Jr., J. A.; Peralta, J. E.; Ogliaro, F.; Bearpark, M.; Heyd, J. J.; Brothers, E.; Kudin, K. N.; Staroverov, V. N.; Keith, T.; Kobayashi, R.; Normand, J.; Raghavachari, K.; Rendell, A.; Burant, J. C.; Iyengar, S. S.; Tomasi, J.; Cossi, M.; Rega, N.; Millam, J. M.; Klene, M.; Knox, J. E.; Cross, J. B.; Bakken, V.; Adamo, C.; Jaramillo, J.; Gomperts, R.; Stratmann, R. E.; Yazyev, O.; Austin, A. J.; Cammi, R.; Pomelli, C.; Ochterski, J. W.; Martin, R. L.; Morokuma, K.; Zakrzewski, V. G.; Voth, G. A.; Salvador, P.; Dannenberg, J. J.; Dapprich, S.; Daniels, A. D.; Farkas, O.; Foresman, J. B.; Ortiz, J. V.; Cioslowski, J.; Fox, D. J. *Gaussian, Inc.,* Wallingford CT, **2013**.

(11) Funes-Ardoiz, I.; Paton, R.S., *GoodVibes v1.0.2*, **2017**, https://zenodo.org/record/841362.

(12) Images generated using *Cylview 20*; Legault, C.Y., Université de Sherbrooke, **2020**, www.cylview.org.

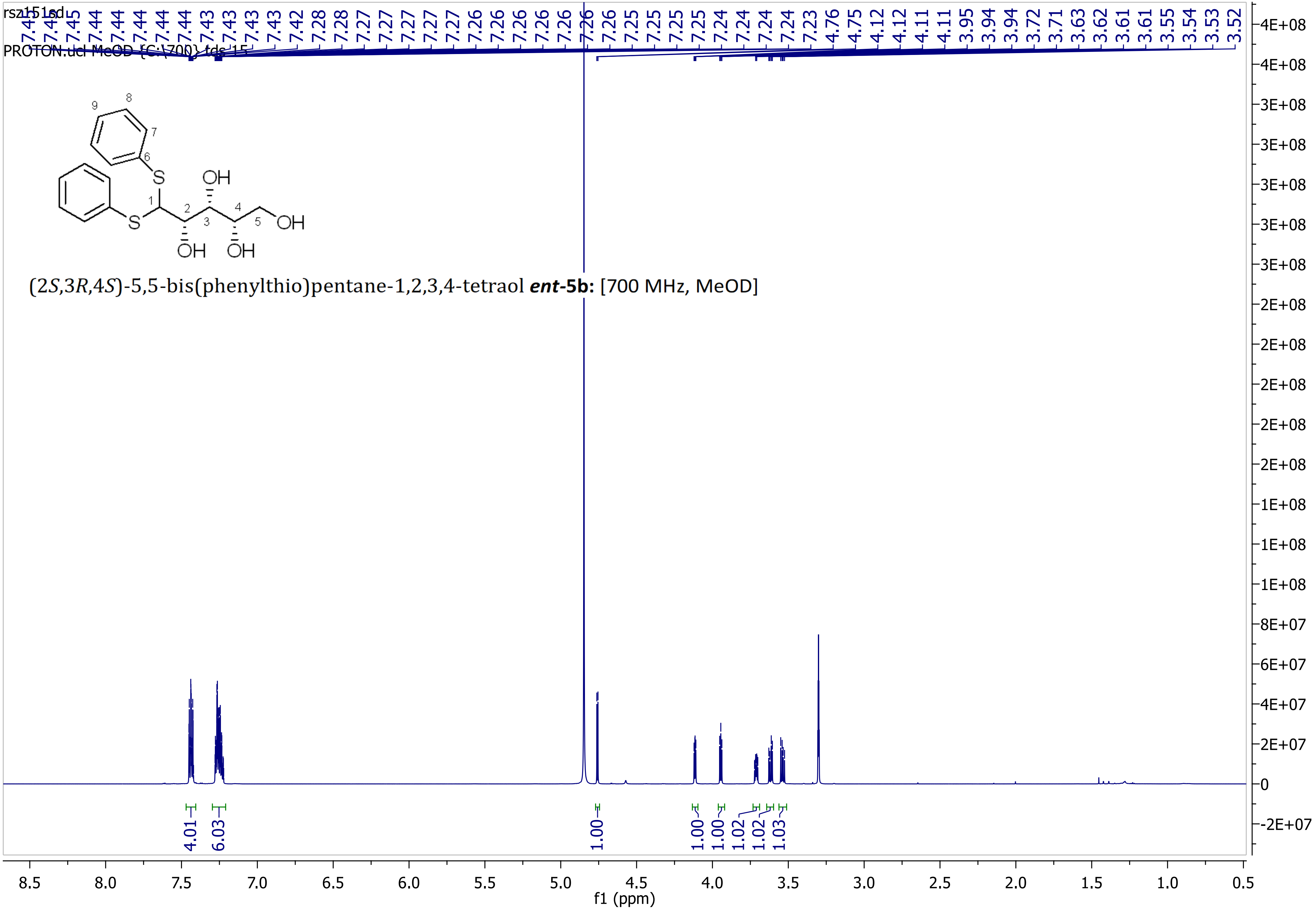


(2*S*,3*R*,4*S*)-5,5-bis(phenylthio)pentane-1,2,3,4-tetraol ***ent*-5b:** [700 MHz, MeOD]

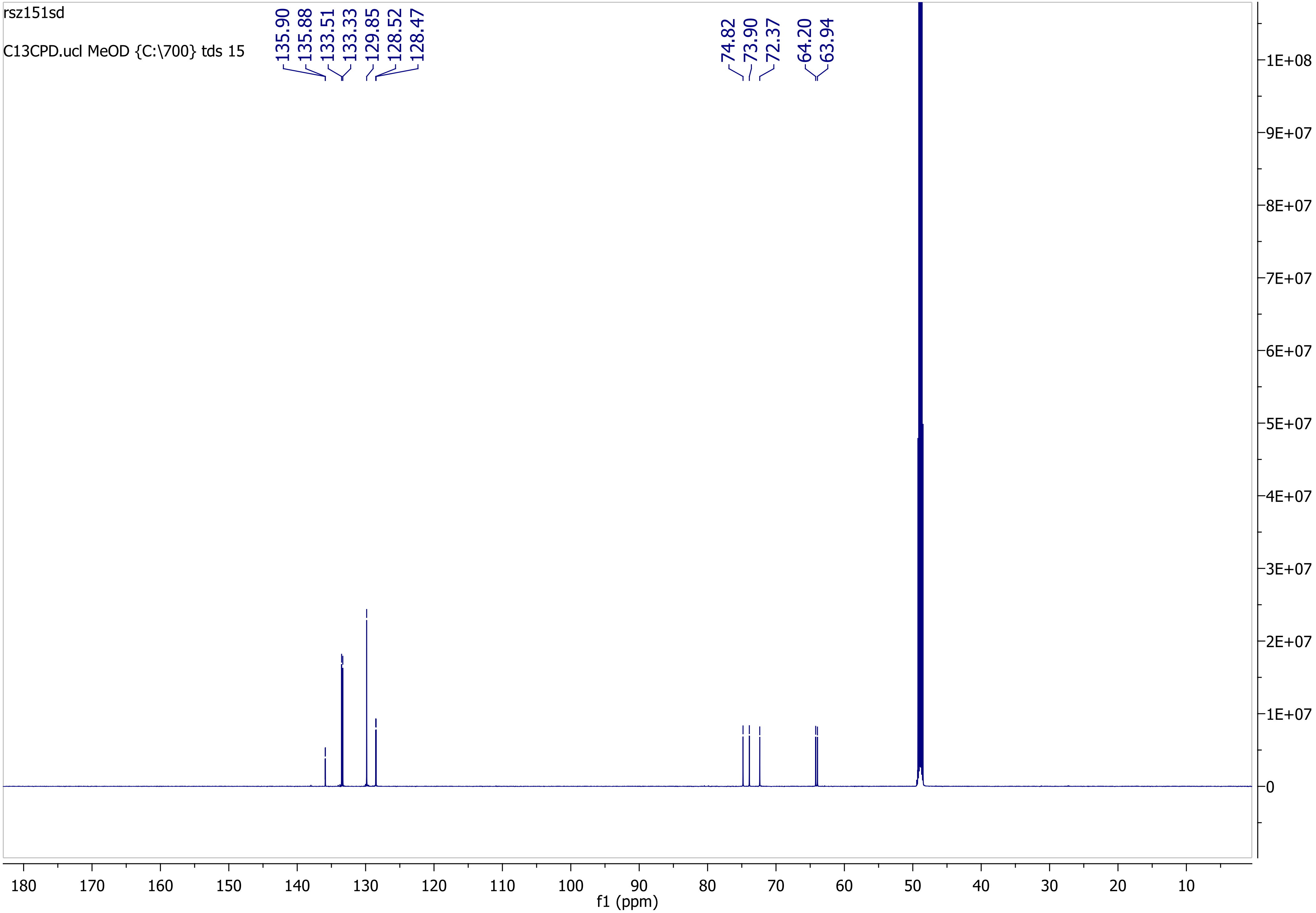

rsz151sd
C13CPD.ucl MeOD {C:\700} tds 15
135.90
135.88
133.51
133.33
129.85
128.52
128.47
74.82
73.90
72.37
64.20
63.94
1E+08
9E+07
8E+07
7E+07
6E+07
5E+07
4E+07
3E+07
2E+07
1E+07
0
180 170 160 150 140 130 120 110 100 90 80 70 60 50 40 30 20 10
f1 (ppm)

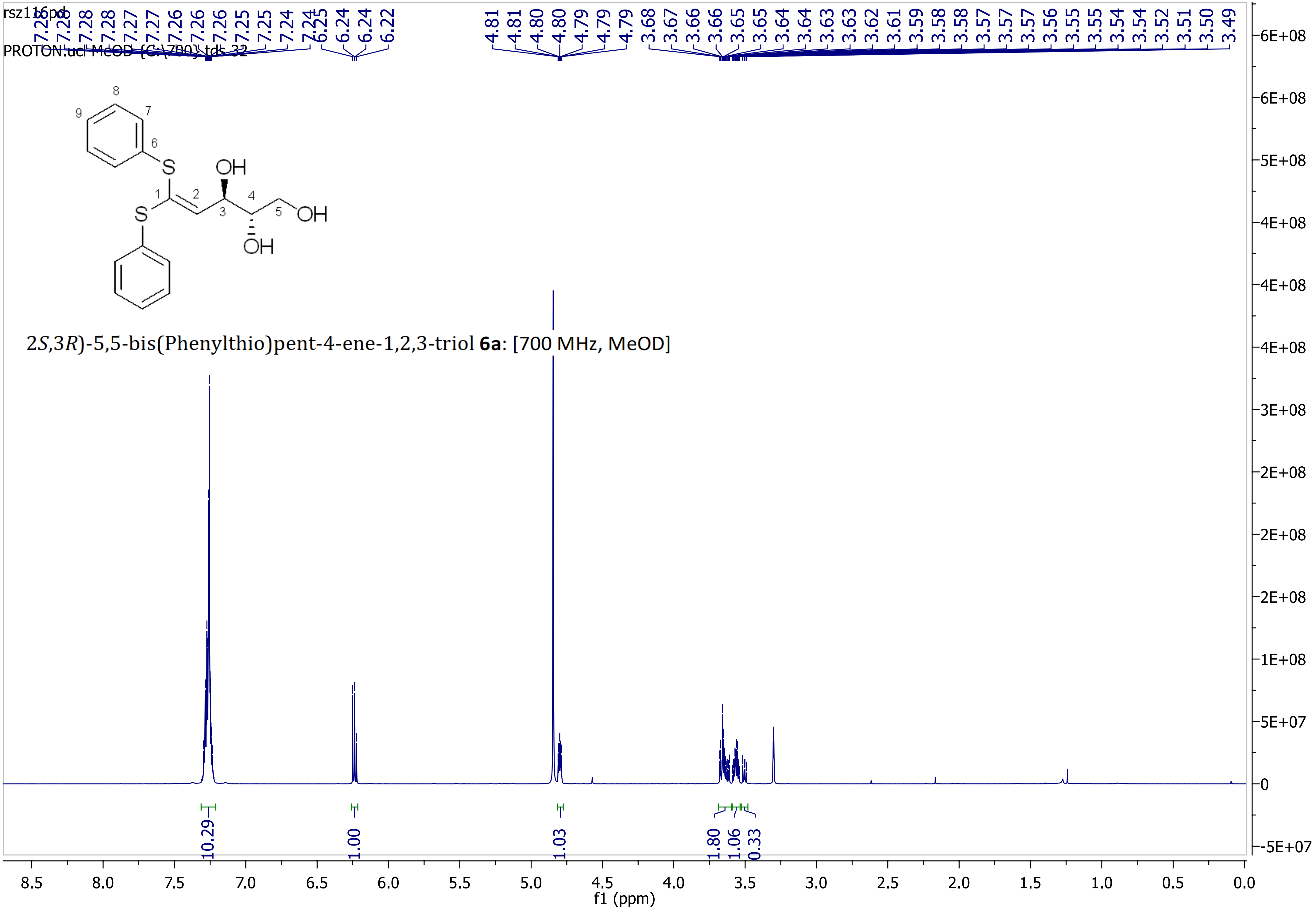
rsz116pd
PROTON.ucl MeOD {C:\700} tds 32
7.28 7.28 7.28 7.27 7.27 7.26 7.26 7.26 7.25 7.25 7.24 7.24
6.25 6.24 6.24 6.22
4.81 4.81 4.80 4.80 4.79 4.79 4.79
3.68 3.67 3.66 3.66 3.65 3.65 3.64 3.64 3.63 3.63 3.62 3.61 3.59 3.58 3.58 3.57 3.57 3.57 3.56 3.55 3.55 3.54 3.54 3.52 3.51 3.50 3.49
S
S
OH
OH
OH
1 2 3 4 5 6 7 8 9
2S,3R)-5,5-bis(Phenylthio)pent-4-ene-1,2,3-triol 6a: [700 MHz, MeOD]
10.29
1.00
1.03
1.80
1.06
0.33
8.5 8.0 7.5 7.0 6.5 6.0 5.5 5.0 4.5 4.0 3.5 3.0 2.5 2.0 1.5 1.0 0.5 0.0
f1 (ppm)
6E+08 6E+08 5E+08 4E+08 4E+08 4E+08 3E+08 2E+08 2E+08 2E+08 1E+08 5E+07 0 -5E+07

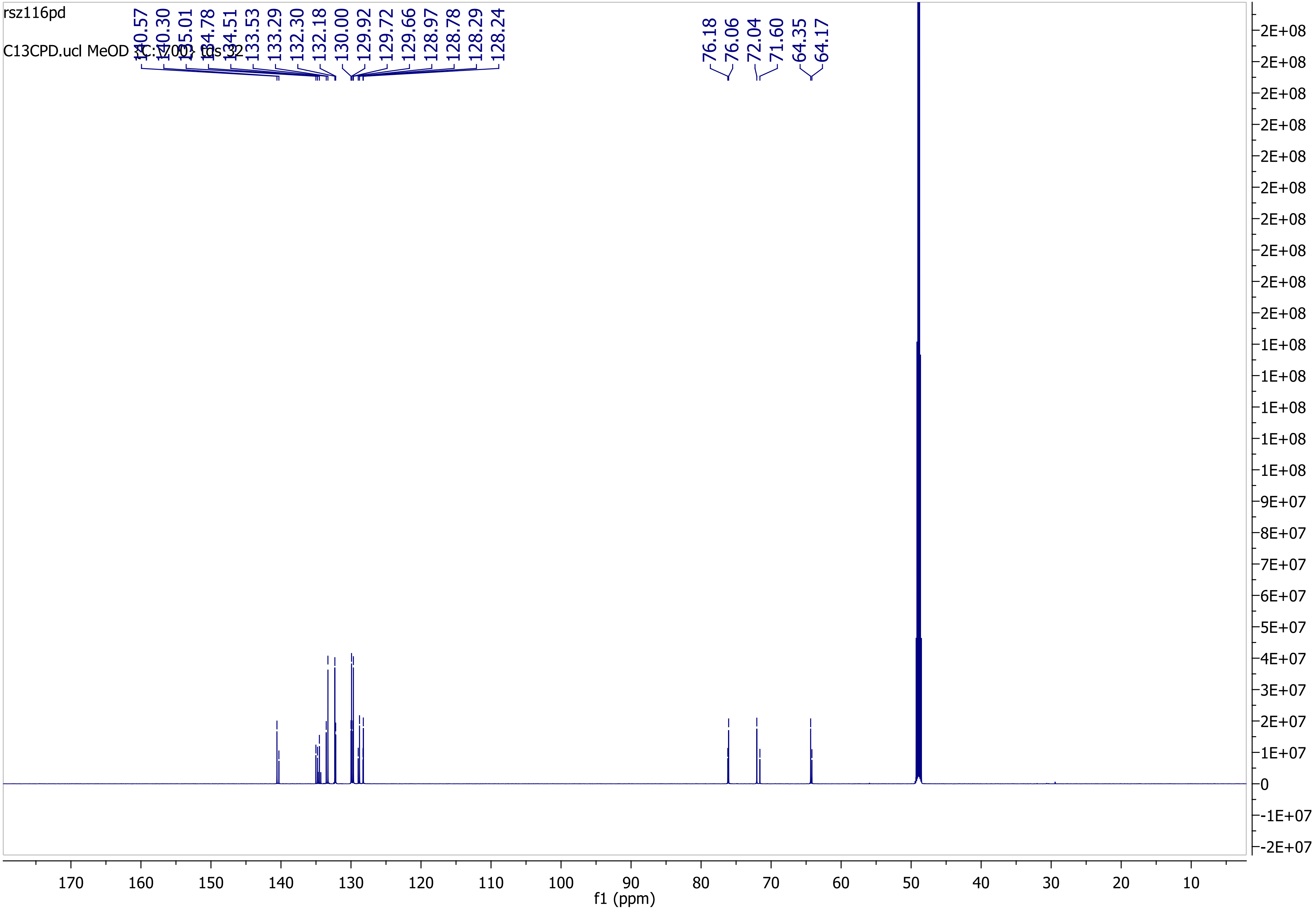
rsz116pd
C13CPD.ucl MeOD {C:\700} tcs 32
140.57
140.30
135.01
134.78
134.51
133.53
133.29
132.30
132.18
130.00
129.92
129.72
129.66
128.97
128.78
128.29
128.24
76.18
76.06
72.04
71.60
64.35
64.17
f1 (ppm)

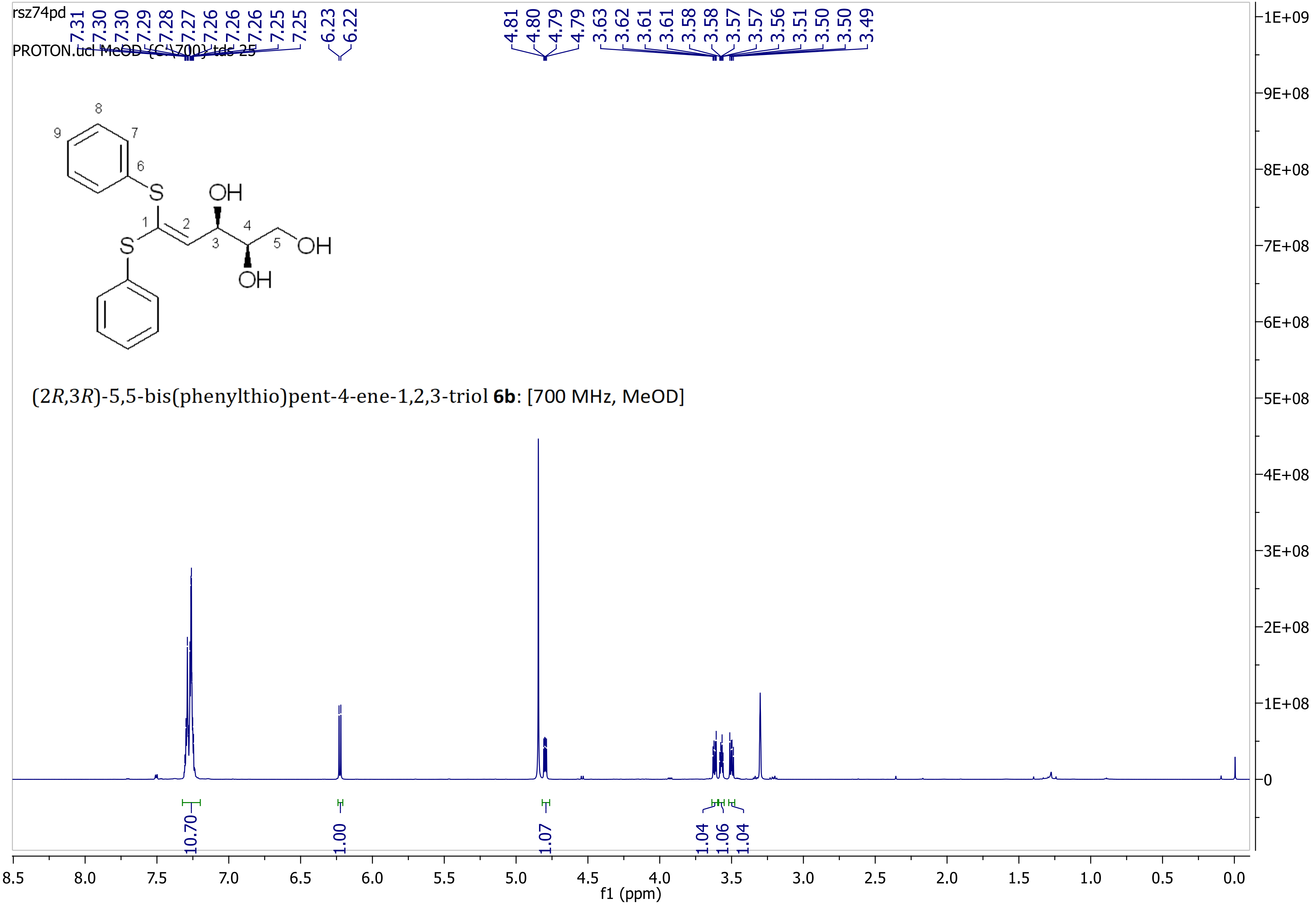


(2*R*,3*R*)-5,5-bis(phenylthio)pent-4-ene-1,2,3-triol **6b**: [700 MHz, MeOD]

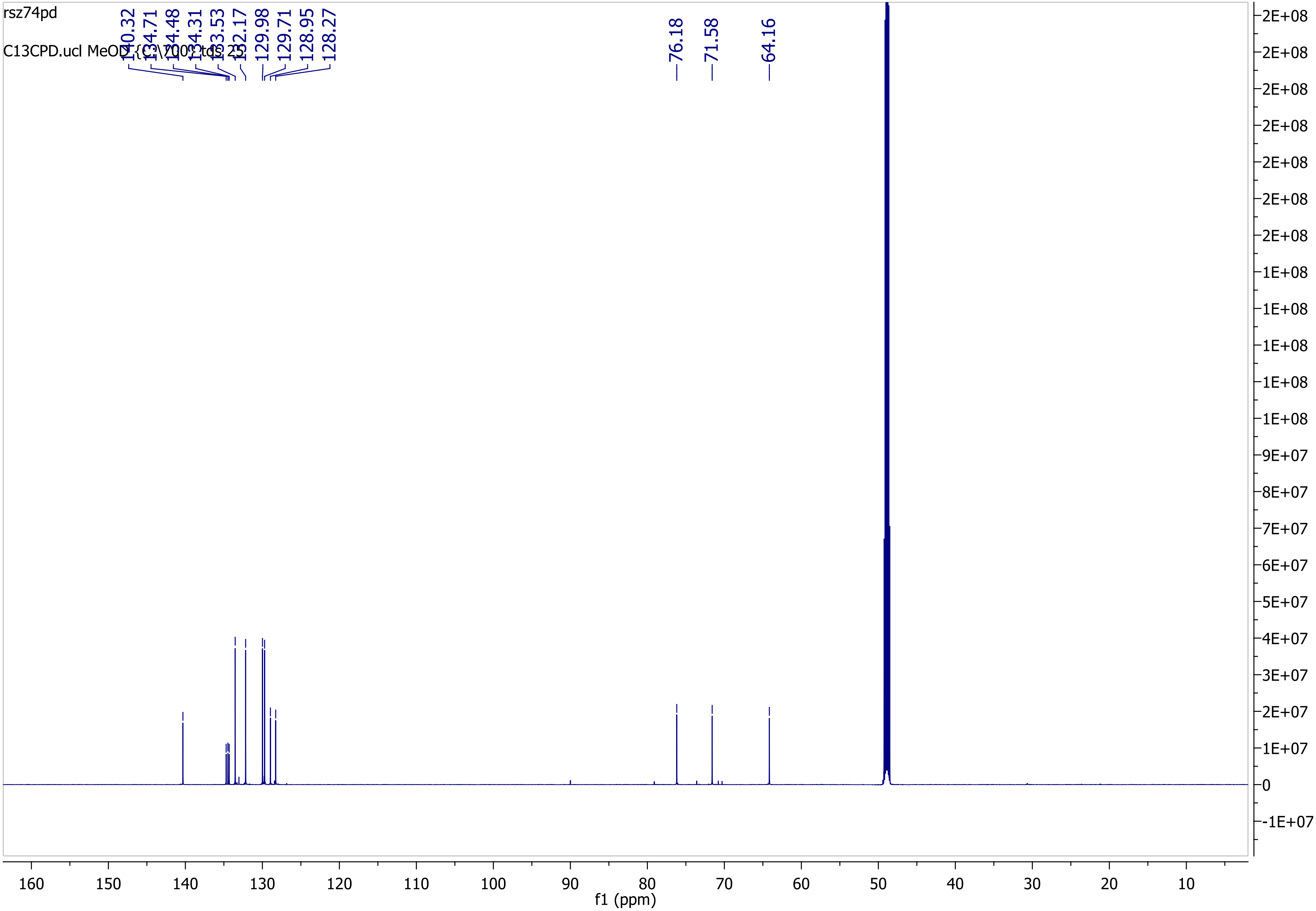
rsz74pd
134.71
133.53
132.17
129.98
129.71
128.95
128.27
76.18
71.58
64.16
f1 (ppm)

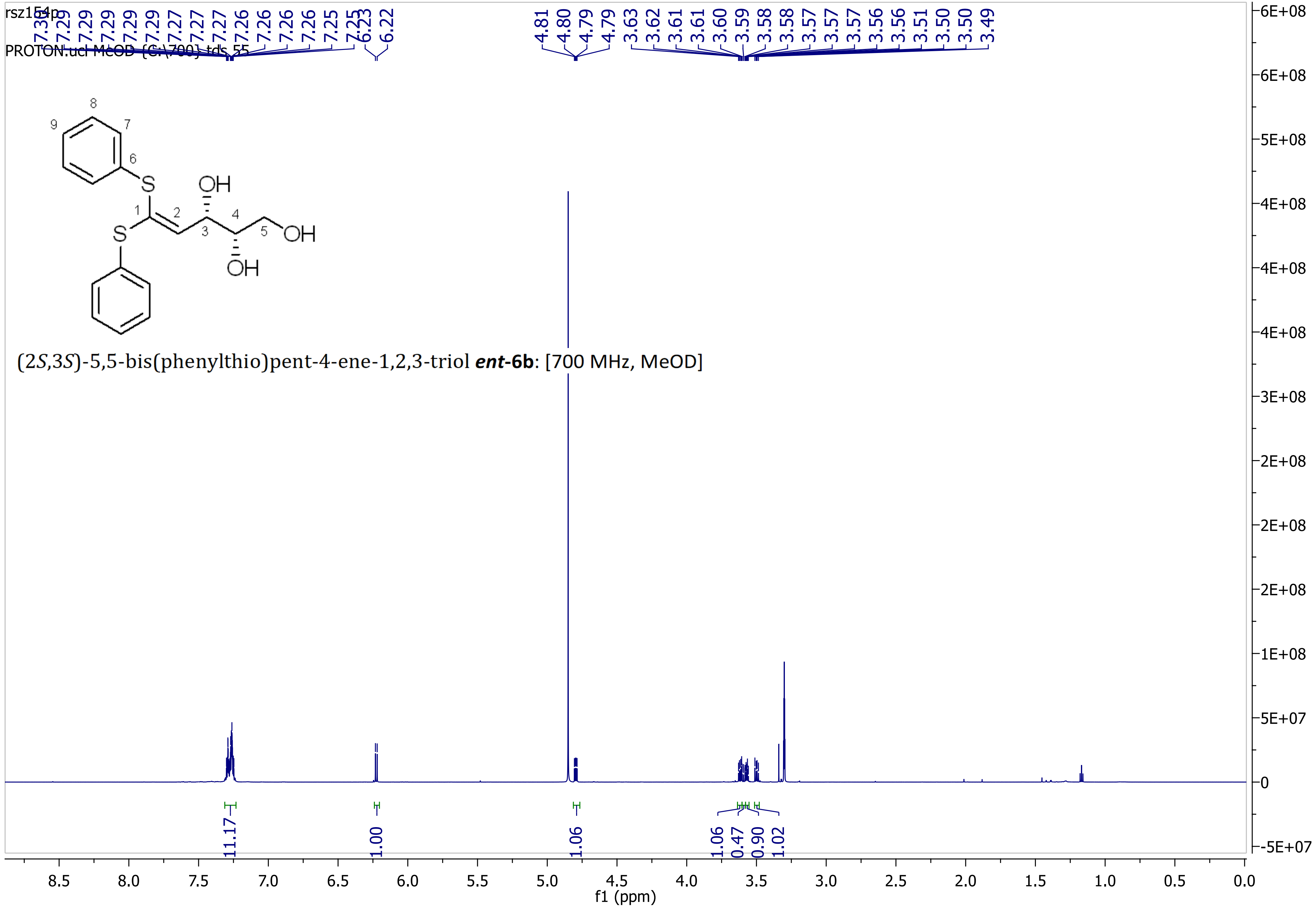


(2*S*,3*S*)-5,5-bis(phenylthio)pent-4-ene-1,2,3-triol ***ent*-6b**: [700 MHz, MeOD]

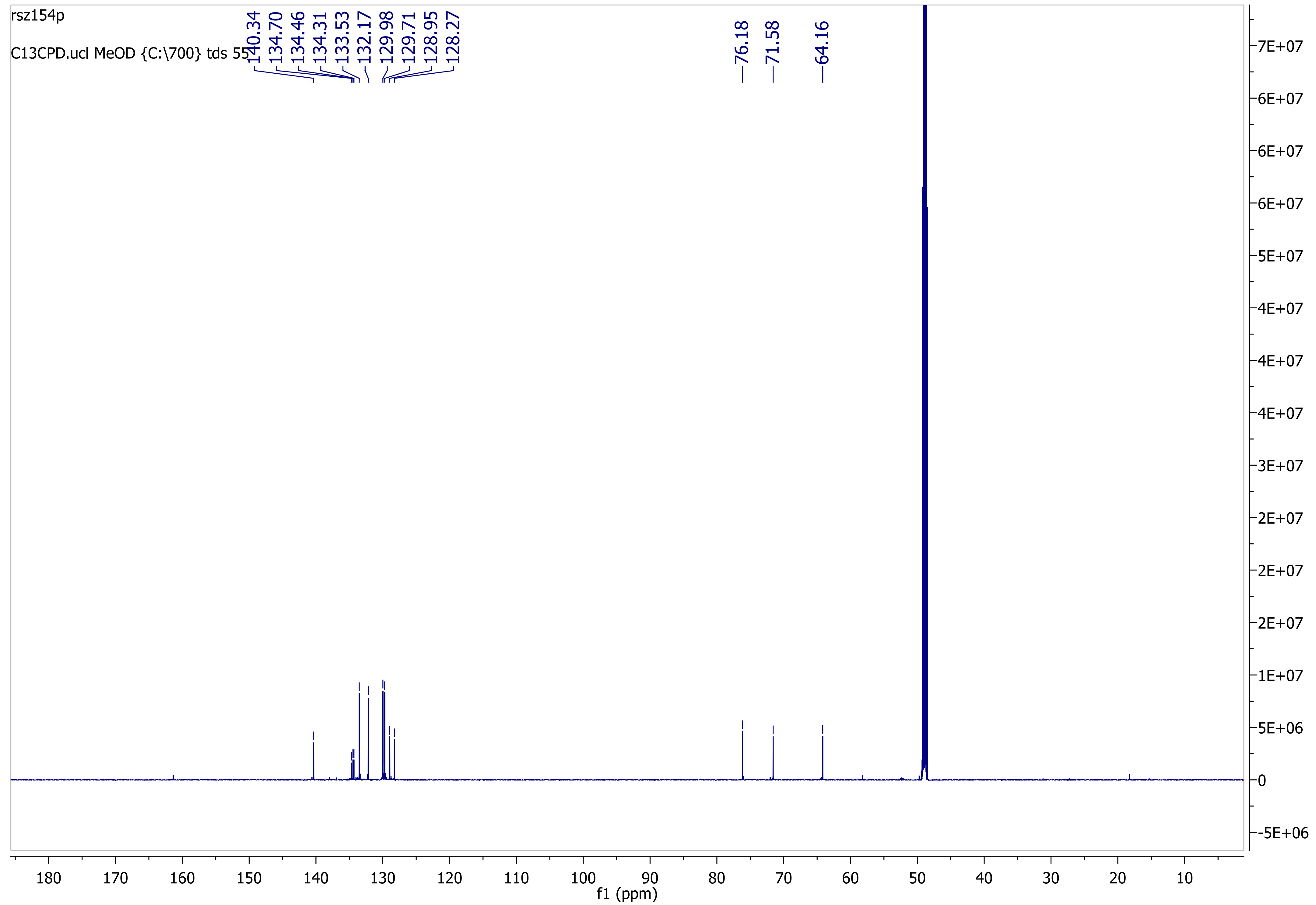
rsz154p
C13CPD.ucl MeOD {C:\700} tds 55
140.34
134.70
134.46
134.31
133.53
132.17
129.98
129.71
128.95
128.27
76.18
71.58
64.16
f1 (ppm)

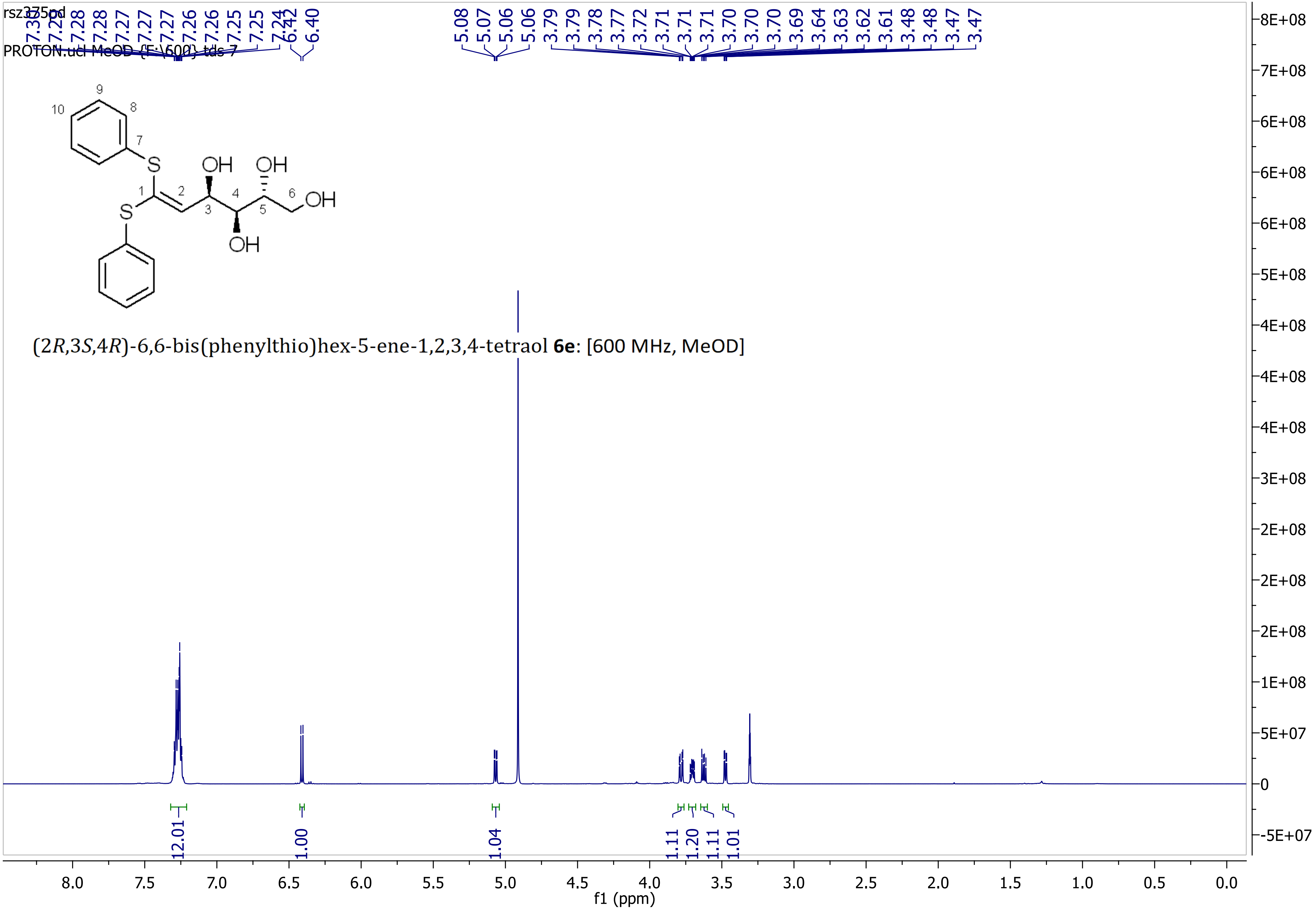
rsz375pd
PROTON.ucl MeOD {E:\600} tds 7
7.28 7.28 7.27 7.27 7.27 7.26 7.26 7.25 7.25 7.24 6.42 6.40
5.08 5.07 5.06 5.06 3.79 3.79 3.78 3.77 3.72 3.71 3.71 3.71 3.70 3.70 3.70 3.69 3.64 3.63 3.62 3.61 3.48 3.48 3.47 3.47
S
S
OH
OH
OH
OH
1 2 3 4 5 6 7 8 9 10
(2R,3S,4R)-6,6-bis(phenylthio)hex-5-ene-1,2,3,4-tetraol 6e: [600 MHz, MeOD]
12.01
1.00
1.04
1.11
1.20
1.11
1.01
8.0 7.5 7.0 6.5 6.0 5.5 5.0 4.5 4.0 3.5 3.0 2.5 2.0 1.5 1.0 0.5 0.0
f1 (ppm)
8E+08 7E+08 6E+08 6E+08 6E+08 5E+08 4E+08 4E+08 4E+08 3E+08 2E+08 2E+08 2E+08 1E+08 5E+07 0 -5E+07

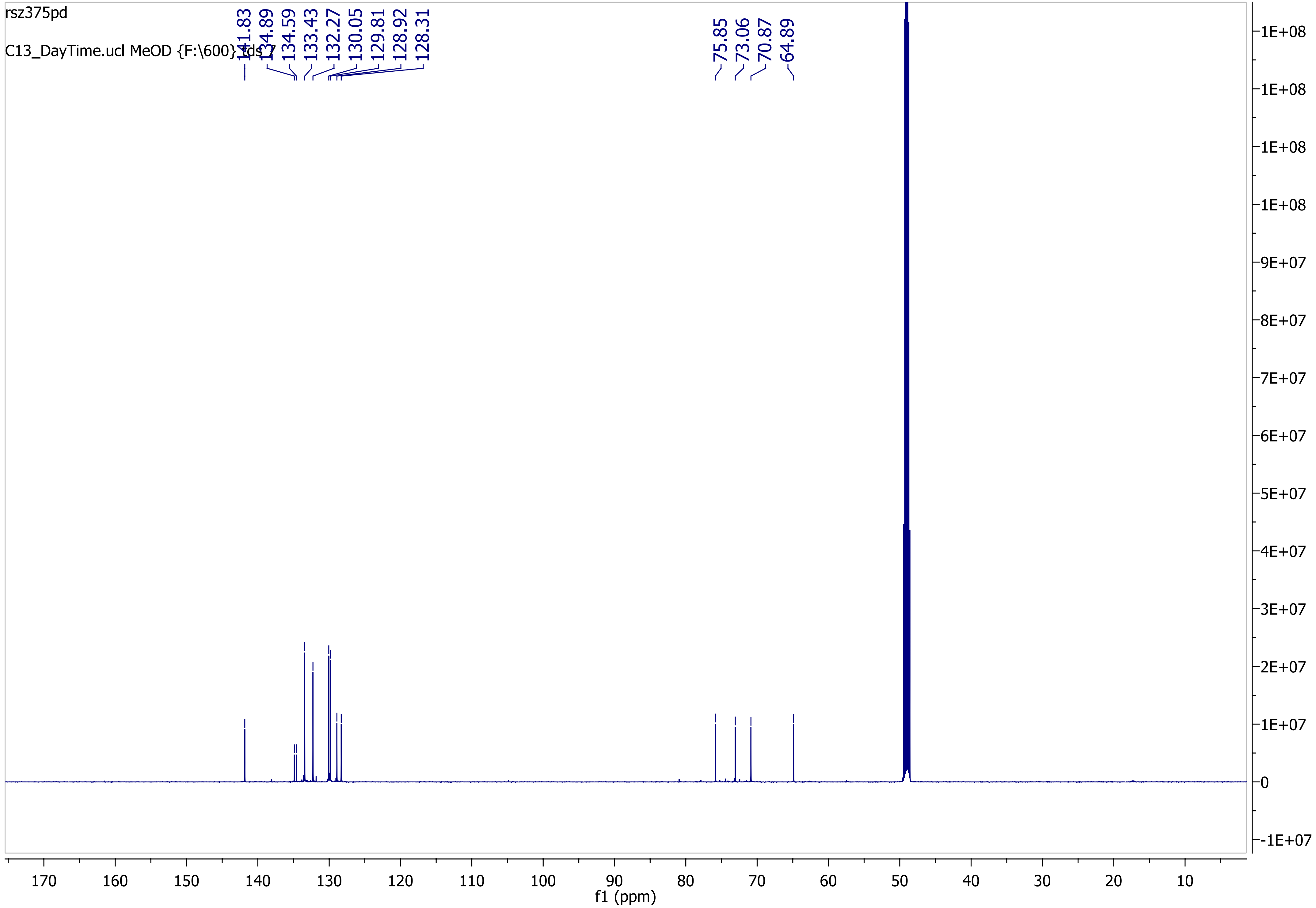

rsz375pd
C13_DayTime.ucl MeOD {F:\600} tds 7
141.83
134.89
134.59
133.43
132.27
130.05
129.81
128.92
128.31
75.85
73.06
70.87
64.89
f1 (ppm)

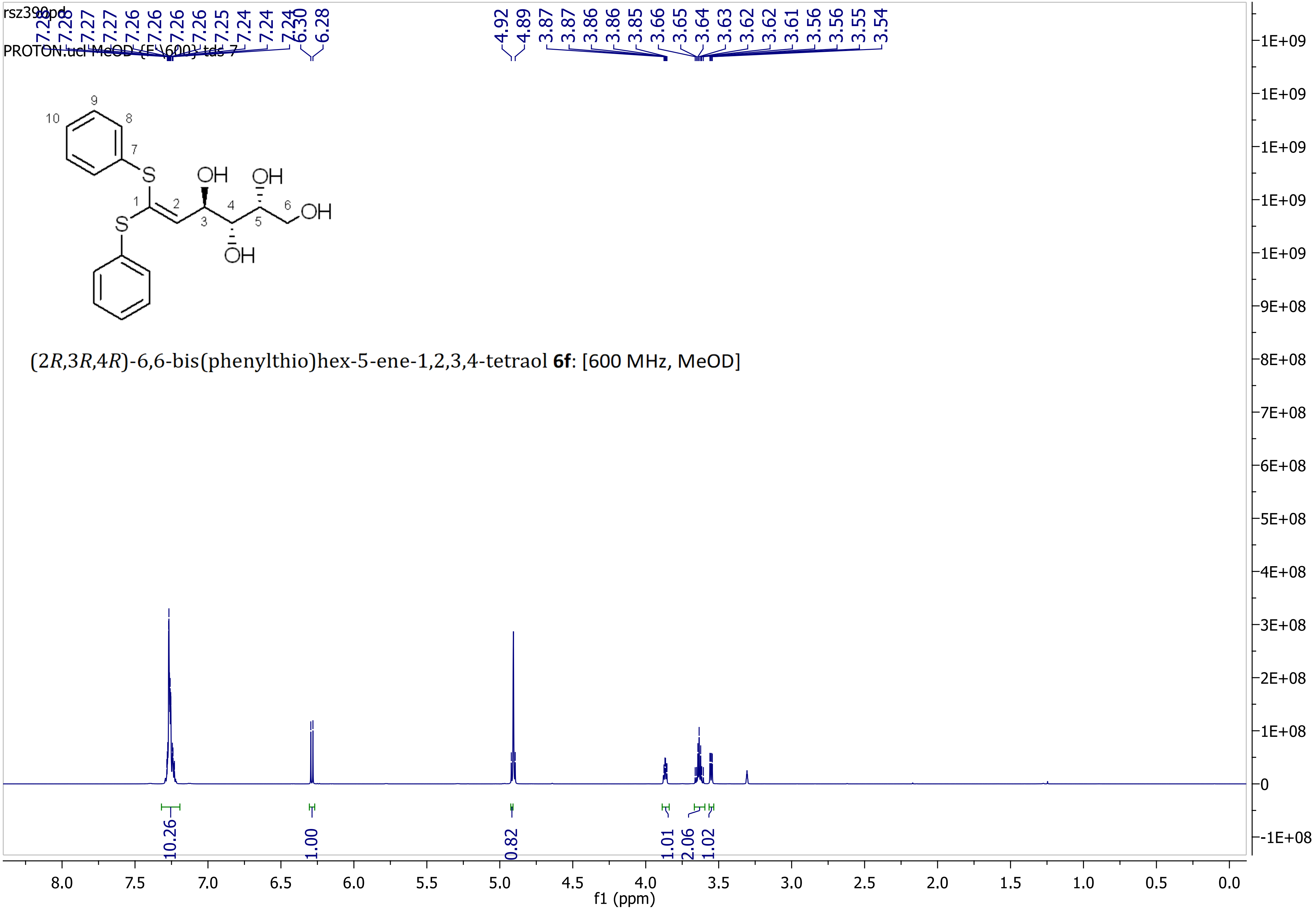
rsz390pd
PROTON.ucl MeOD {E:\600} tds 7
7.28 7.28 7.27 7.27 7.26 7.26 7.26 7.26 7.25 7.24 7.24 7.24 6.30 6.28
4.92 4.89 3.87 3.87 3.86 3.86 3.85 3.66 3.65 3.64 3.63 3.62 3.62 3.61 3.56 3.56 3.55 3.54
(2R,3R,4R)-6,6-bis(phenylthio)hex-5-ene-1,2,3,4-tetraol 6f: [600 MHz, MeOD]
10.26
1.00
0.82
1.01
2.06
1.02
f1 (ppm)
8.0 7.5 7.0 6.5 6.0 5.5 5.0 4.5 4.0 3.5 3.0 2.5 2.0 1.5 1.0 0.5 0.0
1E+09
9E+08
8E+08
7E+08
6E+08
5E+08
4E+08
3E+08
2E+08
1E+08
0
-1E+08

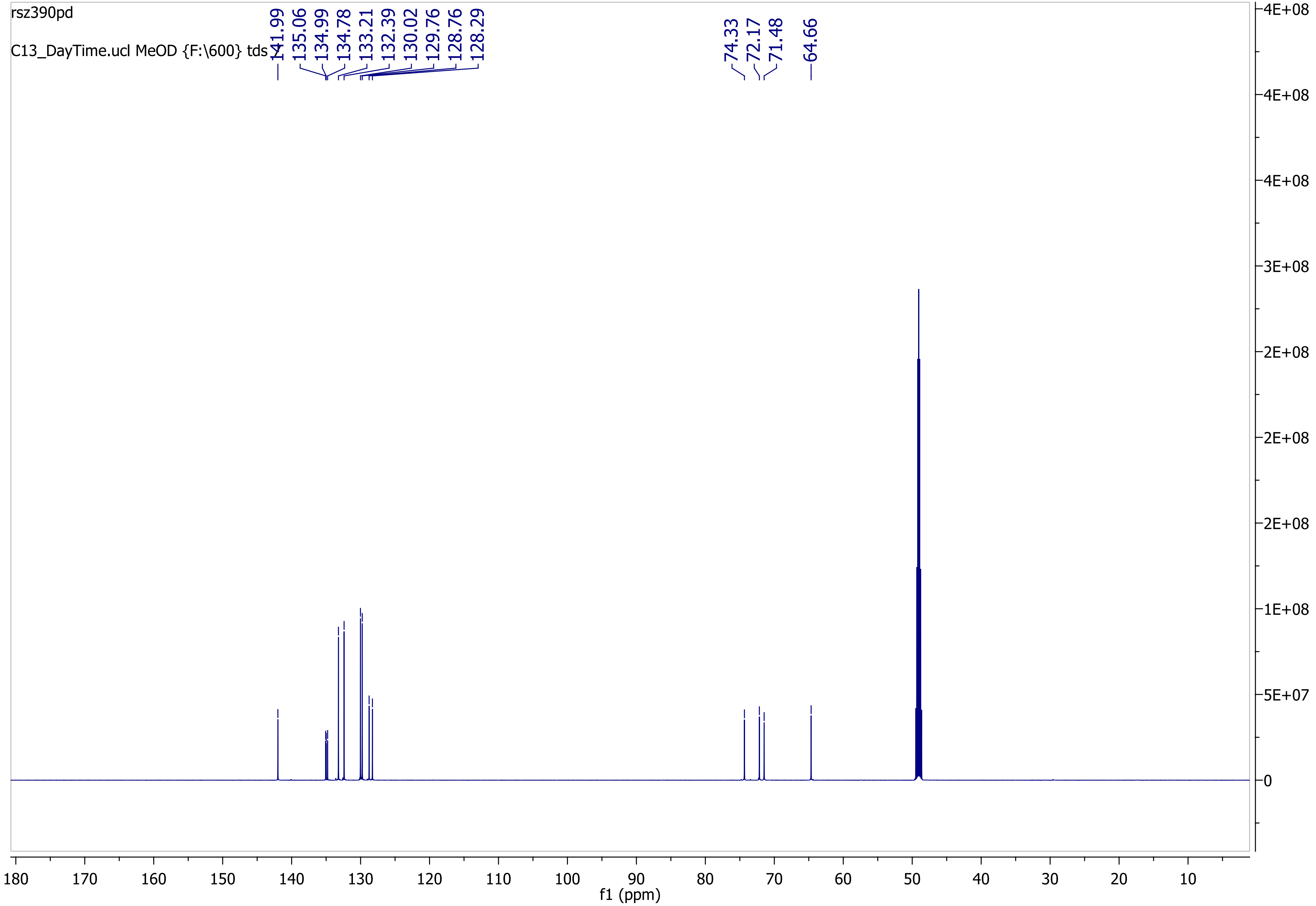
rsz390pd
C13_DayTime.ucl MeOD {F:\600} tds 1
141.99
135.06
134.99
134.78
133.21
132.39
130.02
129.76
128.76
128.29
74.33
72.17
71.48
64.66
f1 (ppm)

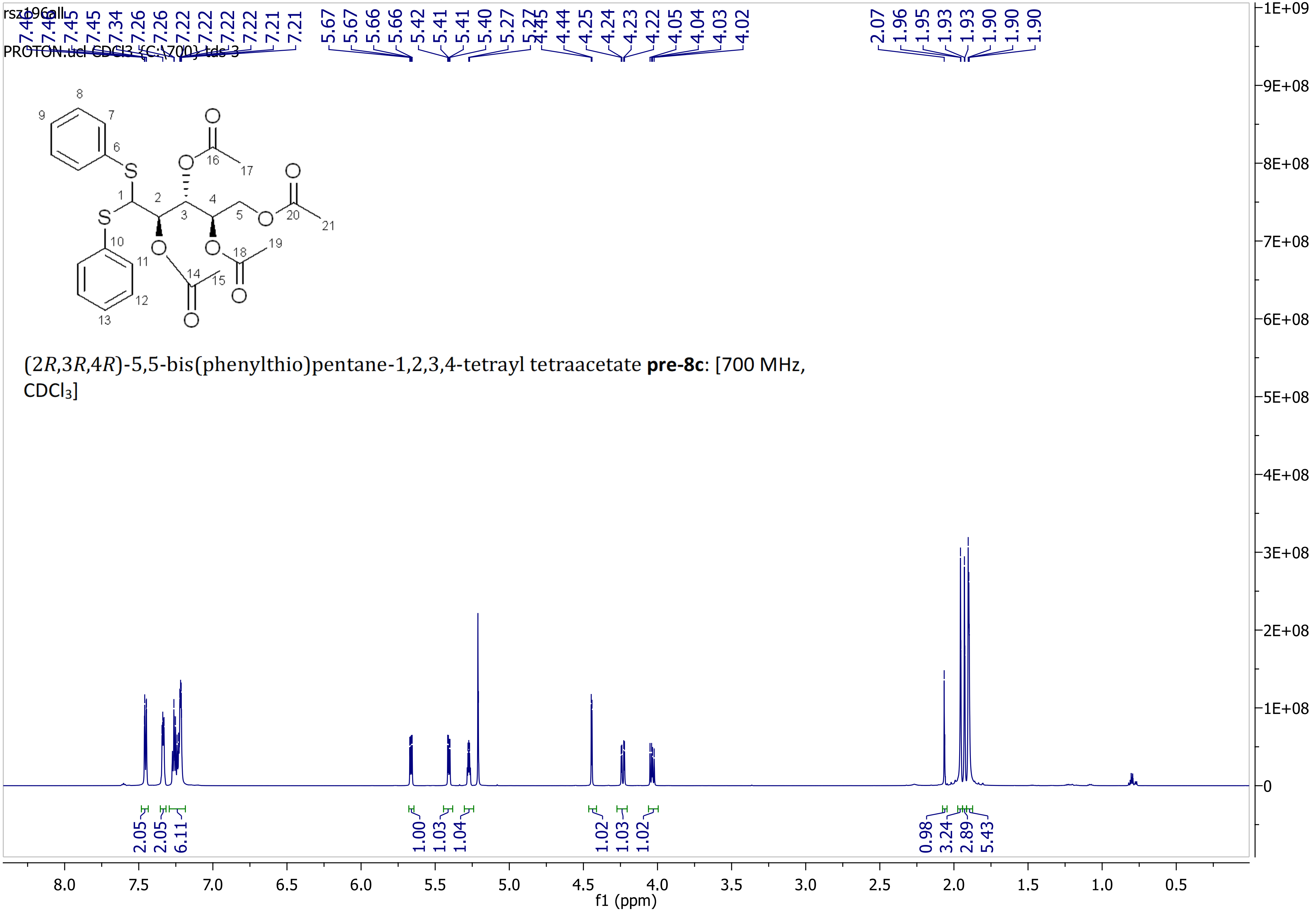


(2*R*,3*R*,4*R*)-5,5-bis(phenylthio)pentane-1,2,3,4-tetrayl tetraacetate **pre-8c**: [700 MHz, $CDCl_3$]

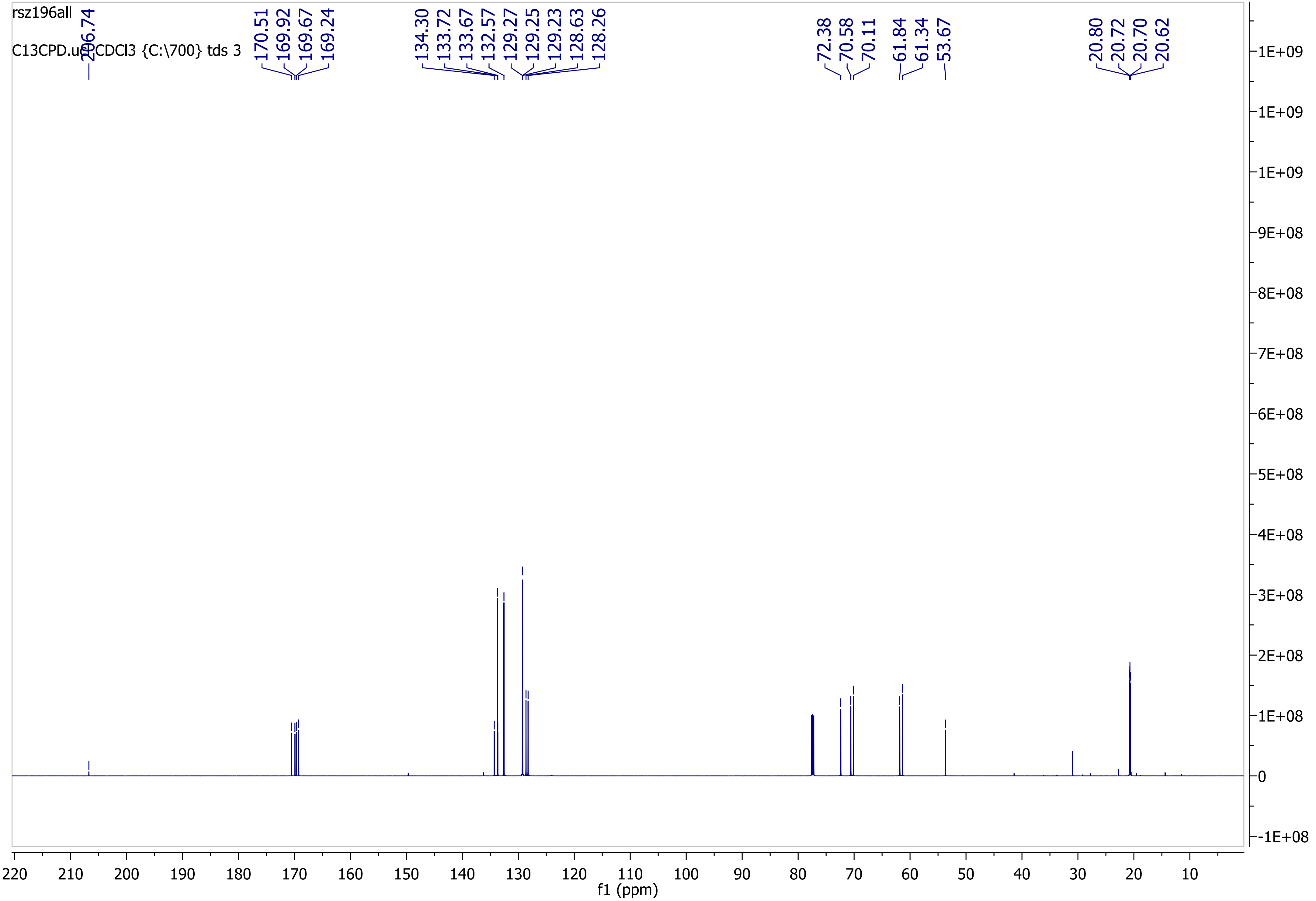

rsz196all
C13CPD.u CDCl3 {C:\700} tds 3
206.74
170.51
169.92
169.67
169.24
134.30
133.72
133.67
132.57
129.27
129.25
129.23
128.63
128.26
72.38
70.58
70.11
61.84
61.34
53.67
20.80
20.72
20.70
20.62
f1 (ppm)

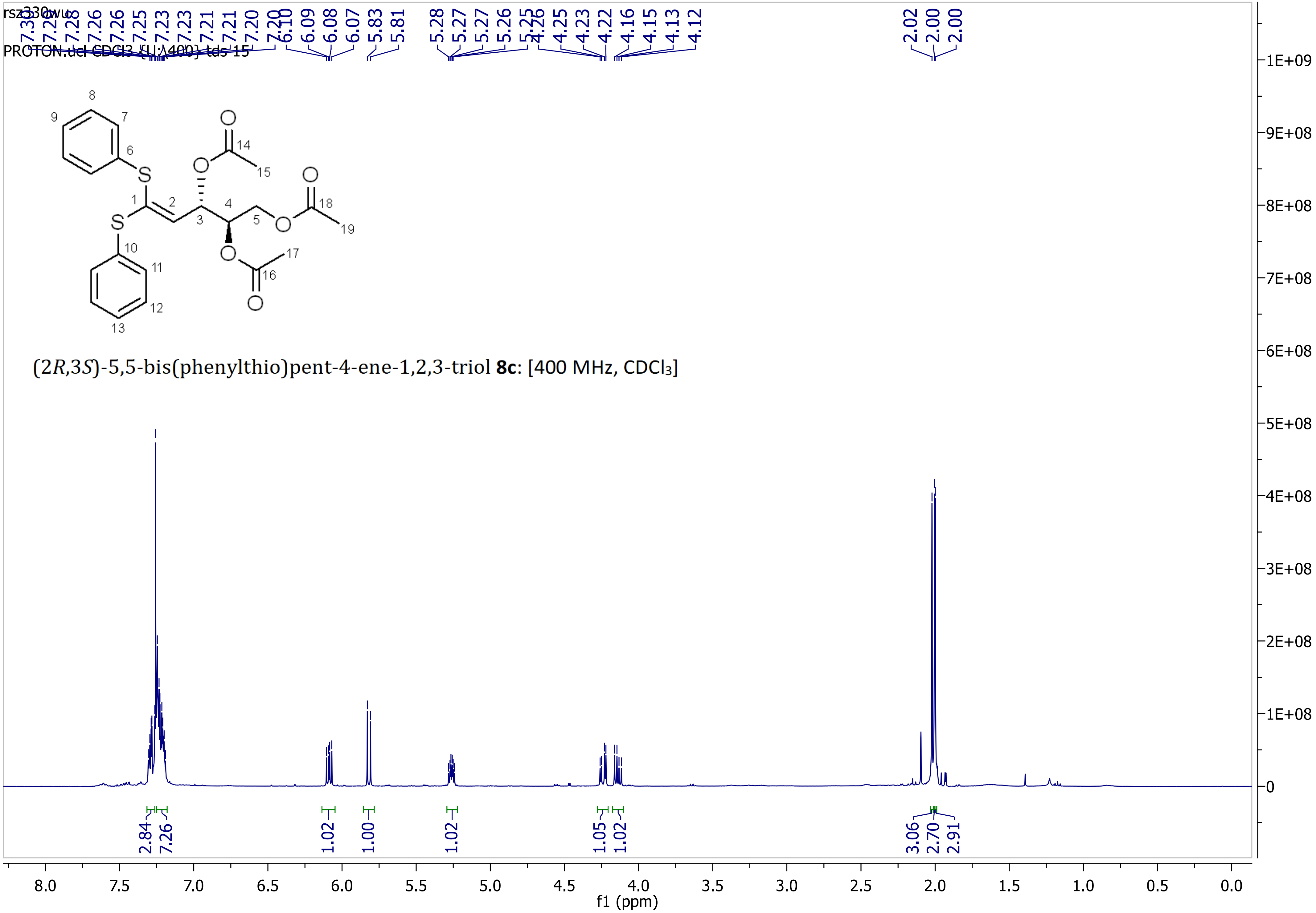


(2*R*,3*S*)-5,5-bis(phenylthio)pent-4-ene-1,2,3-triol **8c**: [400 MHz, $CDCl_3$]

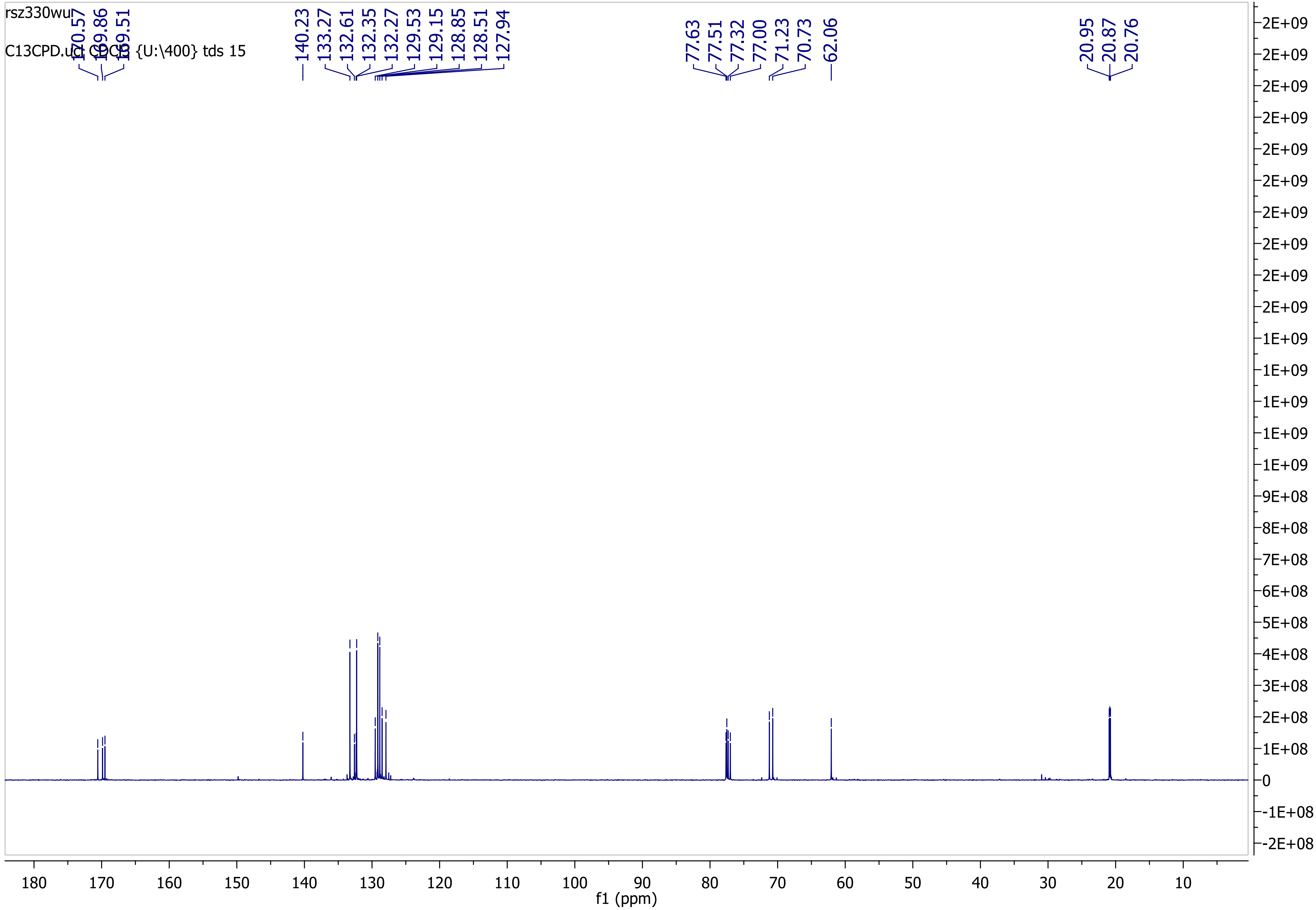
rsz330wu
C13CPD.ucl CDCl3 {U:\400} tds 15
170.57
169.86
169.51
140.23
133.27
132.61
132.35
132.27
129.53
129.15
128.85
128.51
127.94
77.63
77.51
77.32
77.00
71.23
70.73
62.06
20.95
20.87
20.76
f1 (ppm)

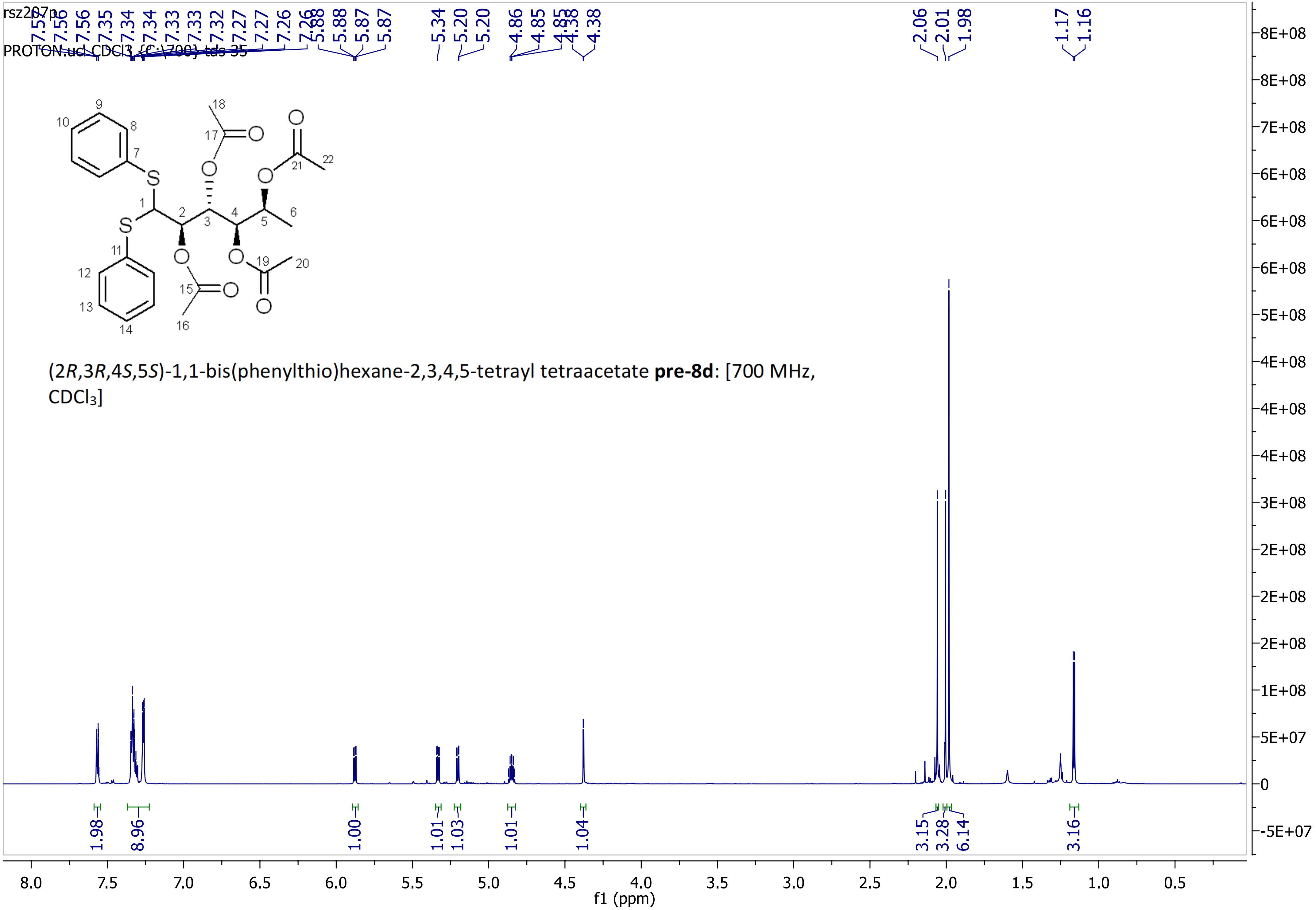
rsz207p
PROTON.ucl CDCl3 {C:\700} tds 35
7.57 7.56 7.56 7.35 7.34 7.34 7.33 7.33 7.32 7.27 7.27 7.26 7.26 5.88 5.88 5.87 5.87 5.34 5.20 5.20 4.86 4.85 4.85 4.38 4.38 2.06 2.01 1.98 1.17 1.16
(2R,3R,4S,5S)-1,1-bis(phenylthio)hexane-2,3,4,5-tetrayl tetraacetate pre-8d: [700 MHz, CDCl3]
1.98 8.96 1.00 1.01 1.03 1.01 1.04 3.15 3.28 6.14 3.16
f1 (ppm)

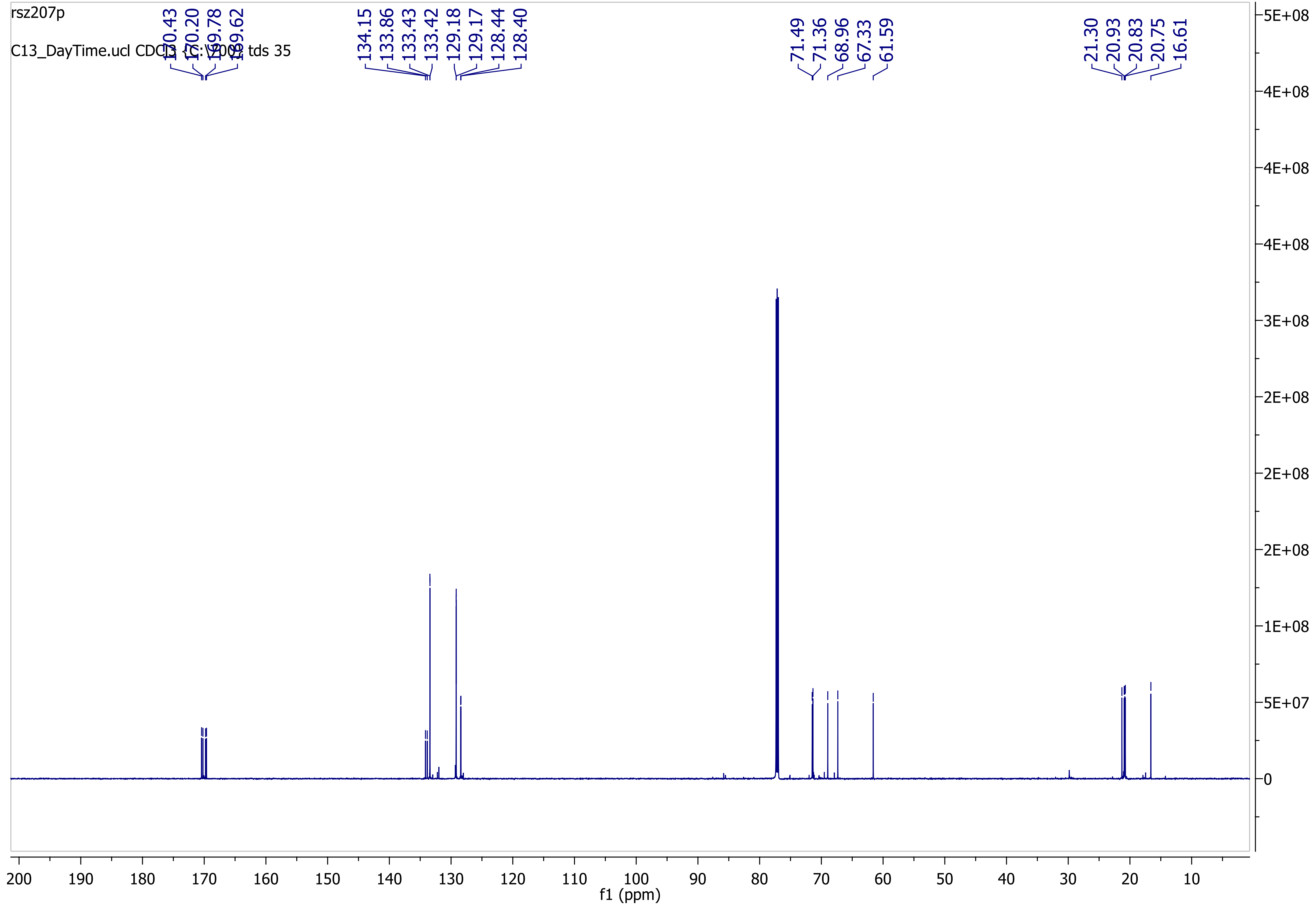
rsz207p
C13_DayTime.ucl CDCl3 {C:\7002} tds 35
170.43
170.20
169.78
169.62
134.15
133.86
133.43
133.42
129.18
129.17
128.44
128.40
71.49
71.36
68.96
67.33
61.59
21.30
20.93
20.83
20.75
16.61
f1 (ppm)

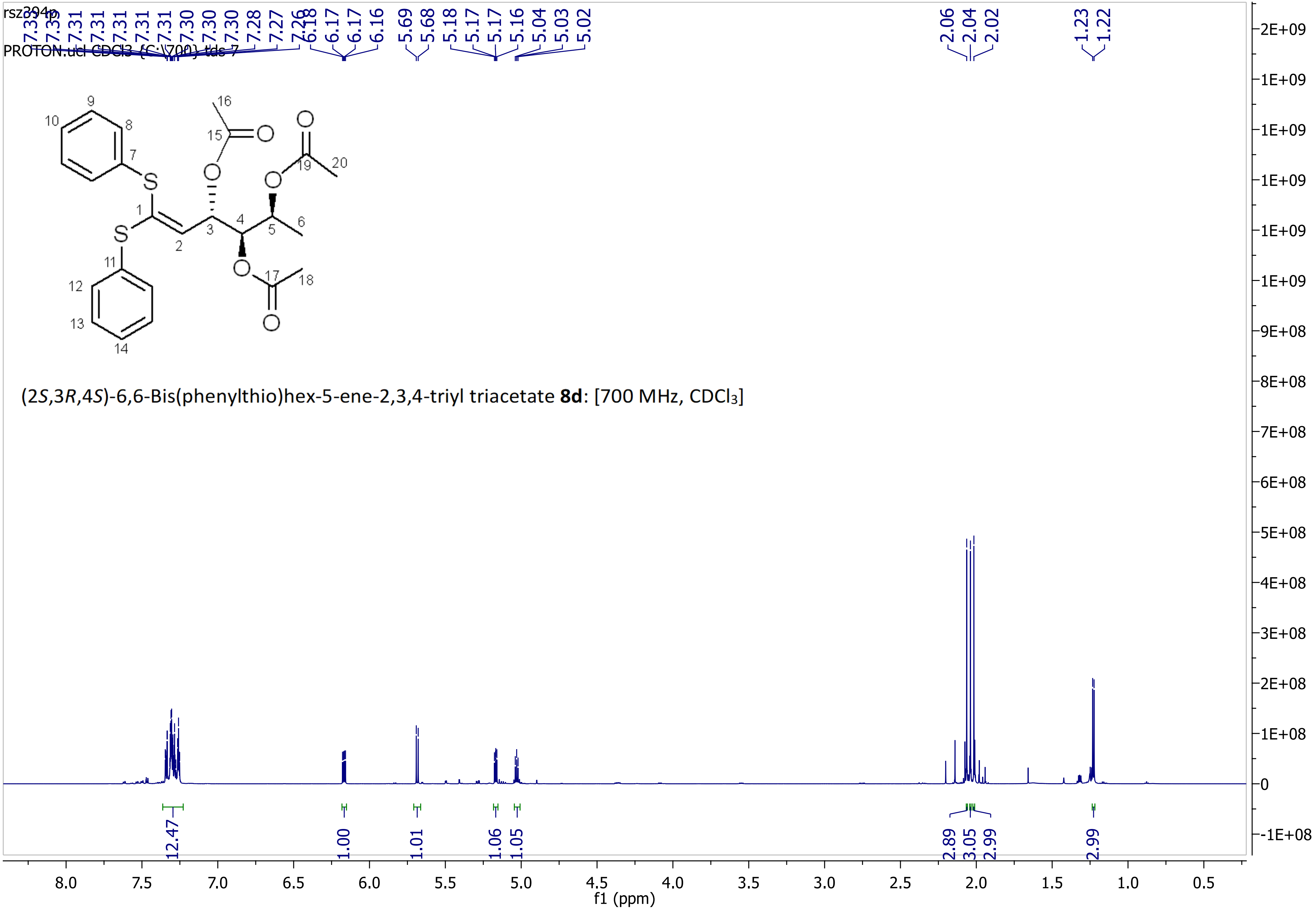


(2*S*,3*R*,4*S*)-6,6-Bis(phenylthio)hex-5-ene-2,3,4-triyl triacetate **8d**: [700 MHz, $CDCl_3$]

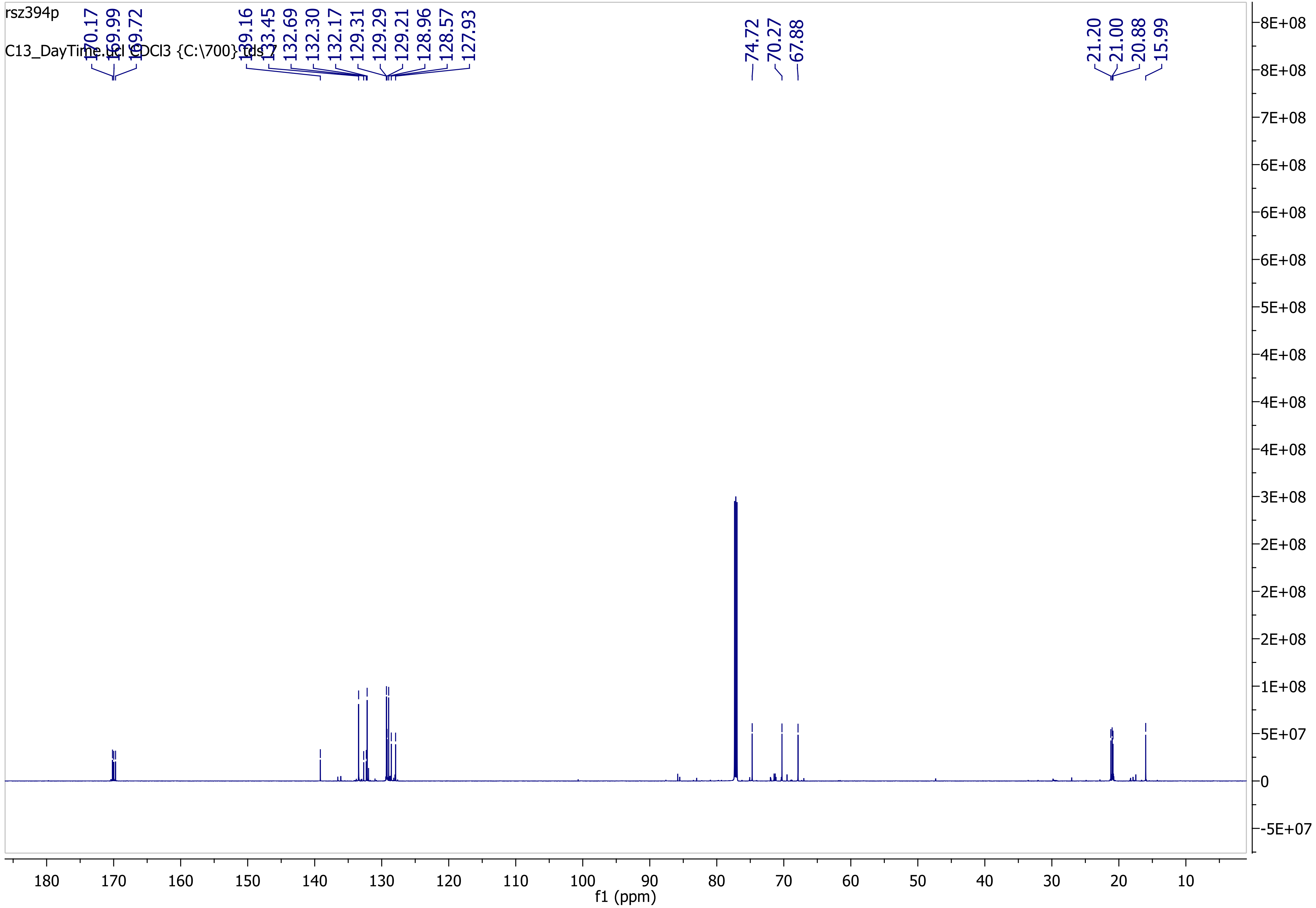

rsz394p
C13_DayTime.ucl CDCl3 {C:\700} tds 7
170.17
169.99
169.72
139.16
133.45
132.69
132.30
132.17
129.31
129.29
129.21
128.96
128.57
127.93
74.72
70.27
67.88
21.20
21.00
20.88
15.99
f1 (ppm)

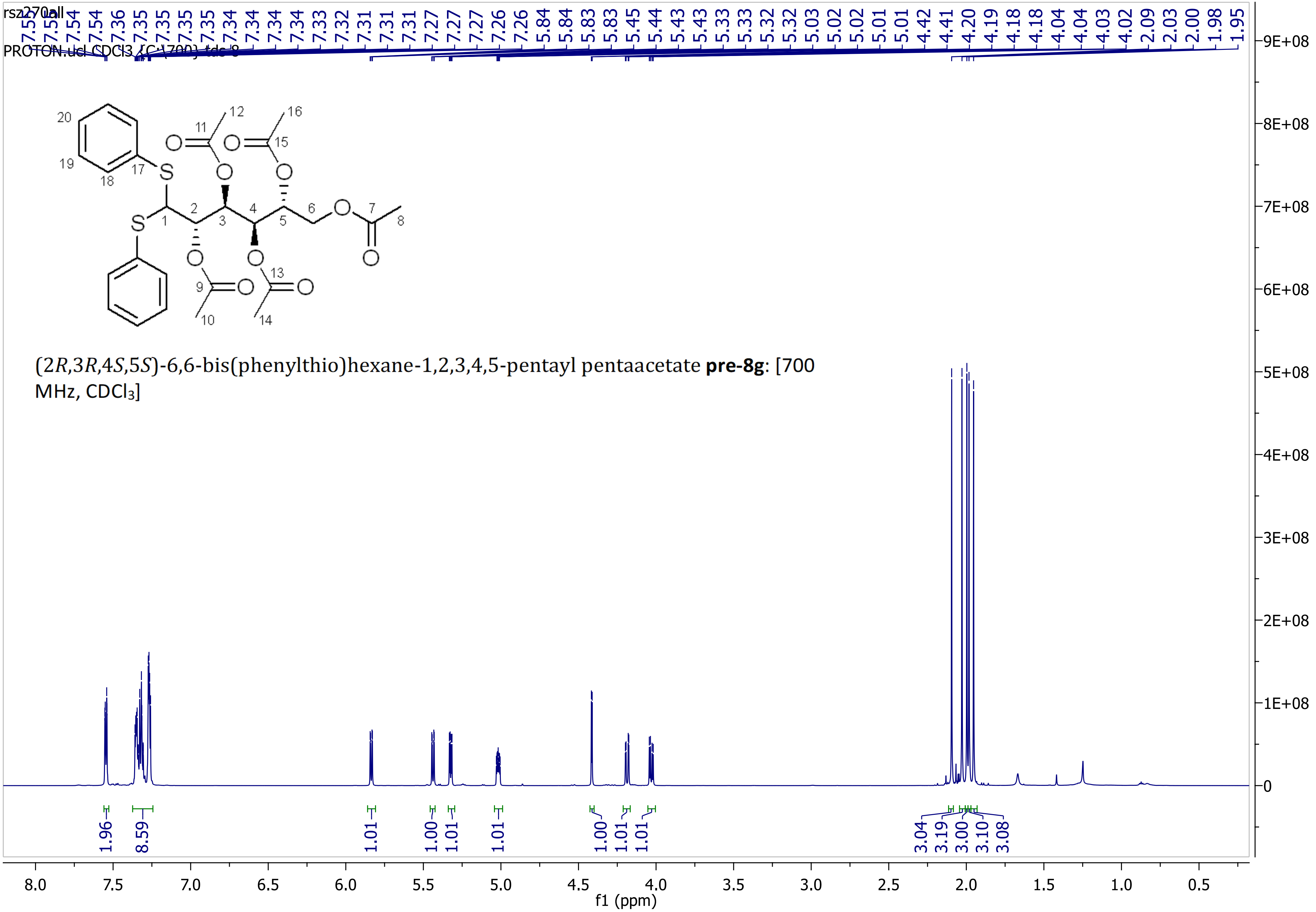


(2*R*,3*R*,4*S*,5*S*)-6,6-bis(phenylthio)hexane-1,2,3,4,5-pentayl pentaacetate **pre-8g**: [700 MHz, $CDCl_3$]

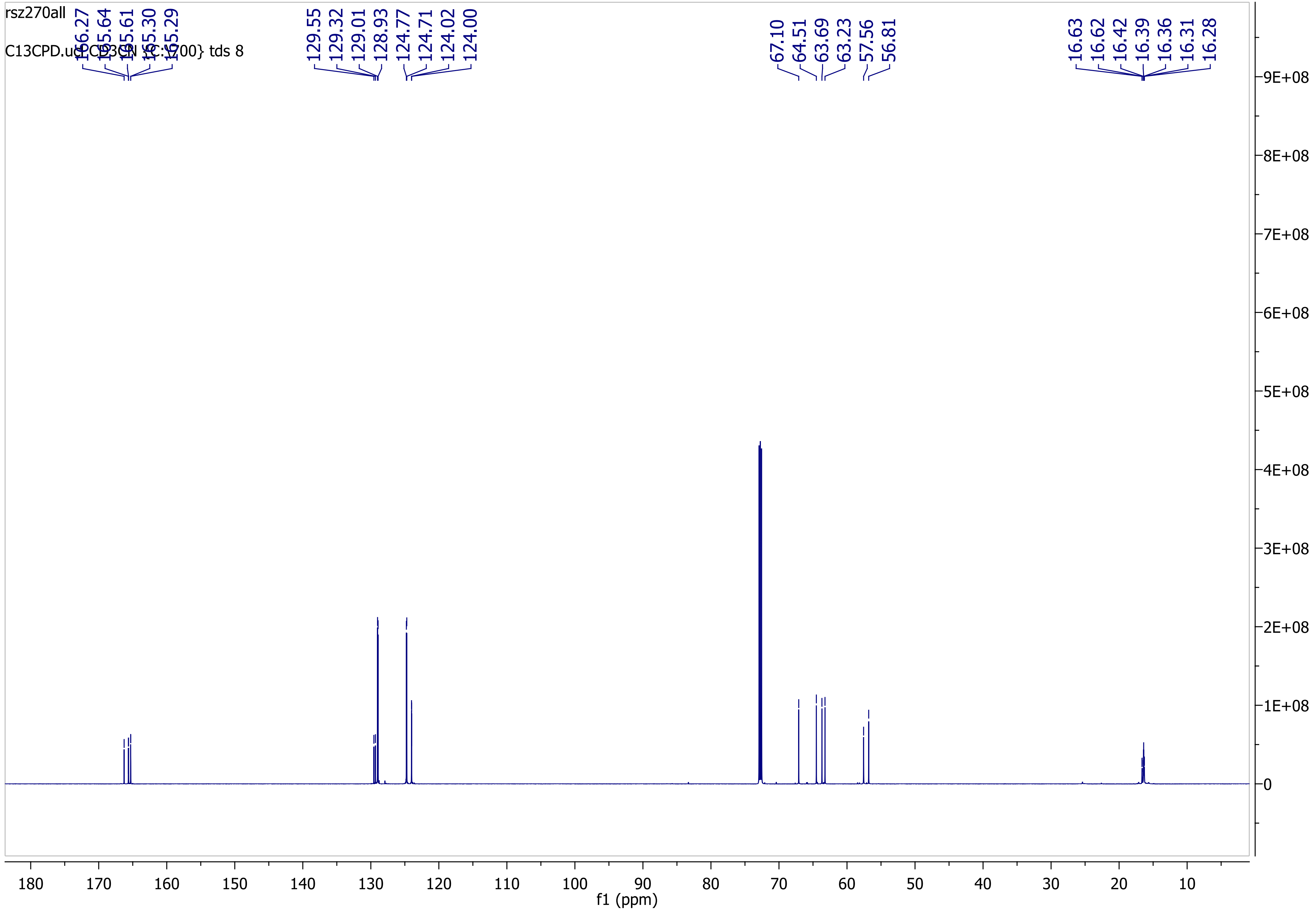
rsz270all
C13CPD.u CD3CN {C:\700} tds 8
166.27
165.64
165.61
165.30
165.29
129.55
129.32
129.01
128.93
124.77
124.71
124.02
124.00
67.10
64.51
63.69
63.23
57.56
56.81
16.63
16.62
16.42
16.39
16.36
16.31
16.28
9E+08
8E+08
7E+08
6E+08
5E+08
4E+08
3E+08
2E+08
1E+08
0
180
170
160
150
140
130
120
110
100
90
80
70
60
50
40
30
20
10
f1 (ppm)

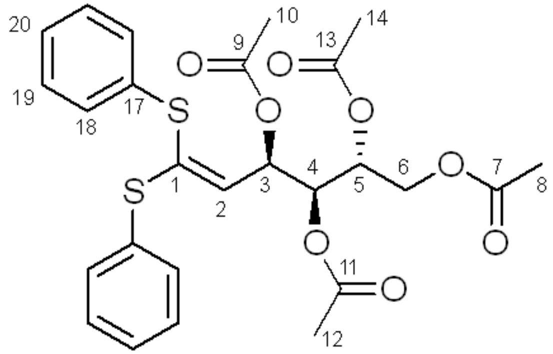


(2*R*,3*S*,4*R*)-6,6-Bis(phenylthio)hex-5-ene-1,2,3,4-tetrayl tetraacetate **8g**: [700 MHz, $CDCl_3$]

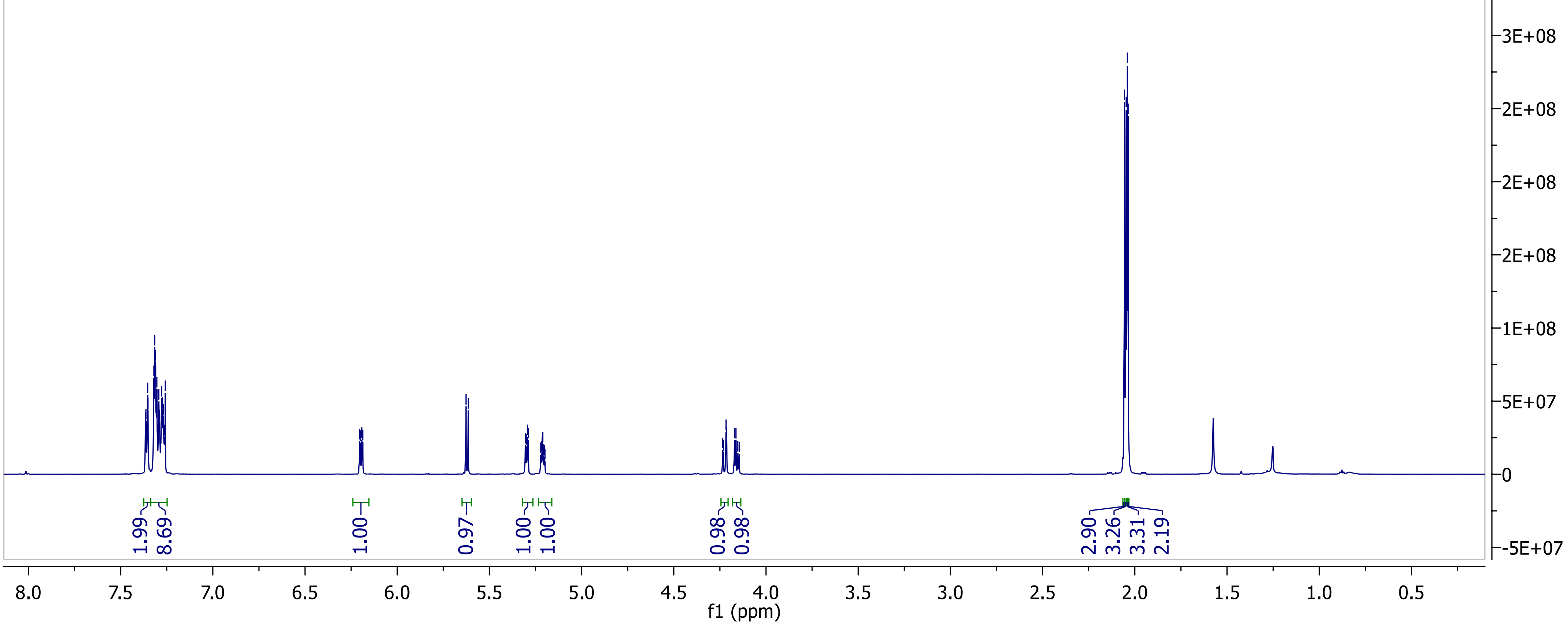

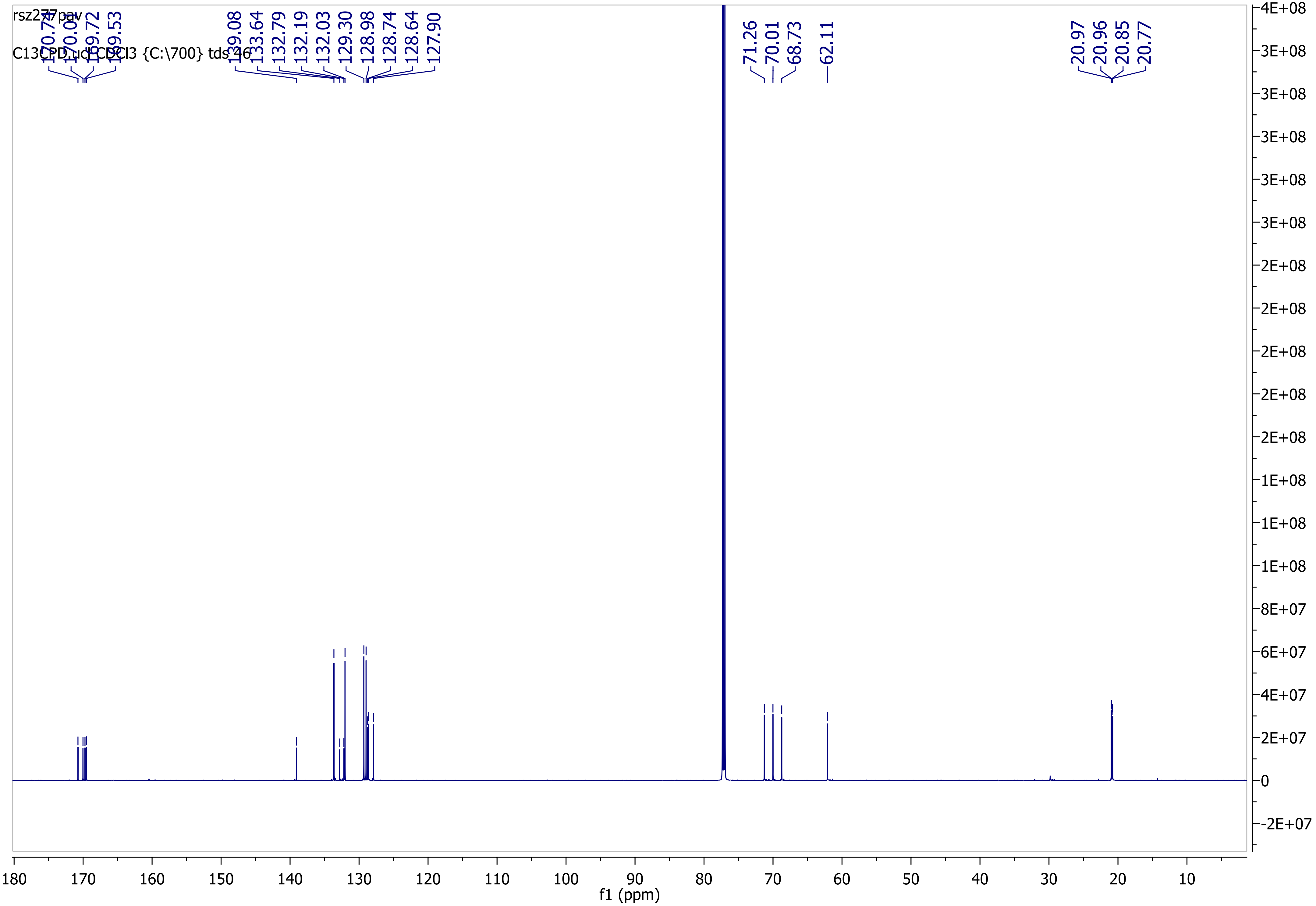

rsz277pav
C13CPD.ucl CDCl3 {C:\700} tds 46
170.74
170.01
169.72
169.53
139.08
133.64
132.79
132.19
132.03
129.30
128.98
128.74
128.64
127.90
71.26
70.01
68.73
62.11
20.97
20.96
20.85
20.77
f1 (ppm)

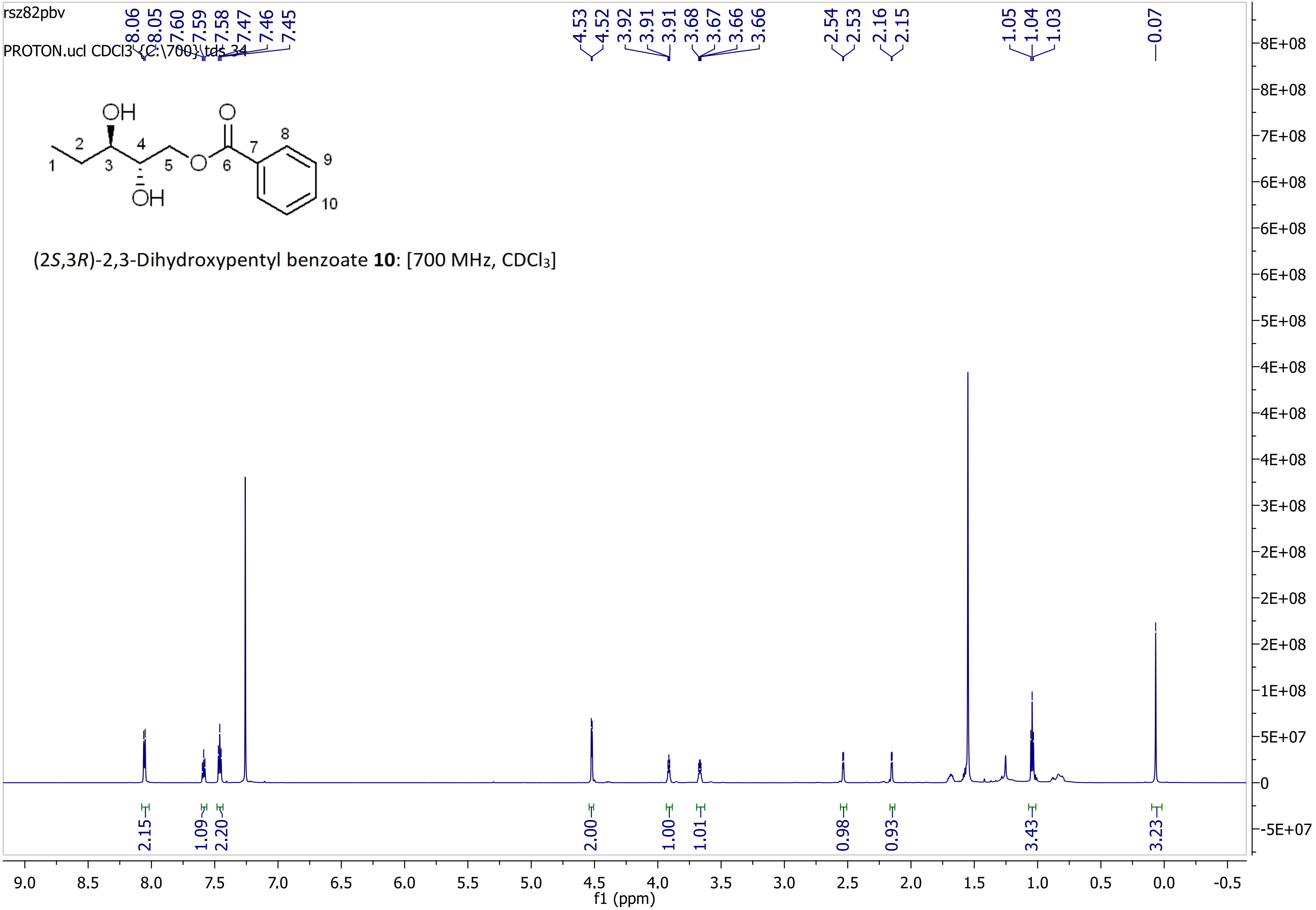
rsz82pbv
PROTON.ucl CDCl3 {C:\700} tds 34
8.06 8.05 7.60 7.59 7.58 7.47 7.46 7.45
4.53 4.52 3.92 3.91 3.91 3.68 3.67 3.66 3.66
2.54 2.53 2.16 2.15
1.05 1.04 1.03
0.07
OH
O
OH
1 2 3 4 5 6 7 8 9 10
(2S,3R)-2,3-Dihydroxypentyl benzoate 10: [700 MHz, CDCl3]
2.15 1.09 2.20 2.00 1.00 1.01 0.98 0.93 3.43 3.23
f1 (ppm)

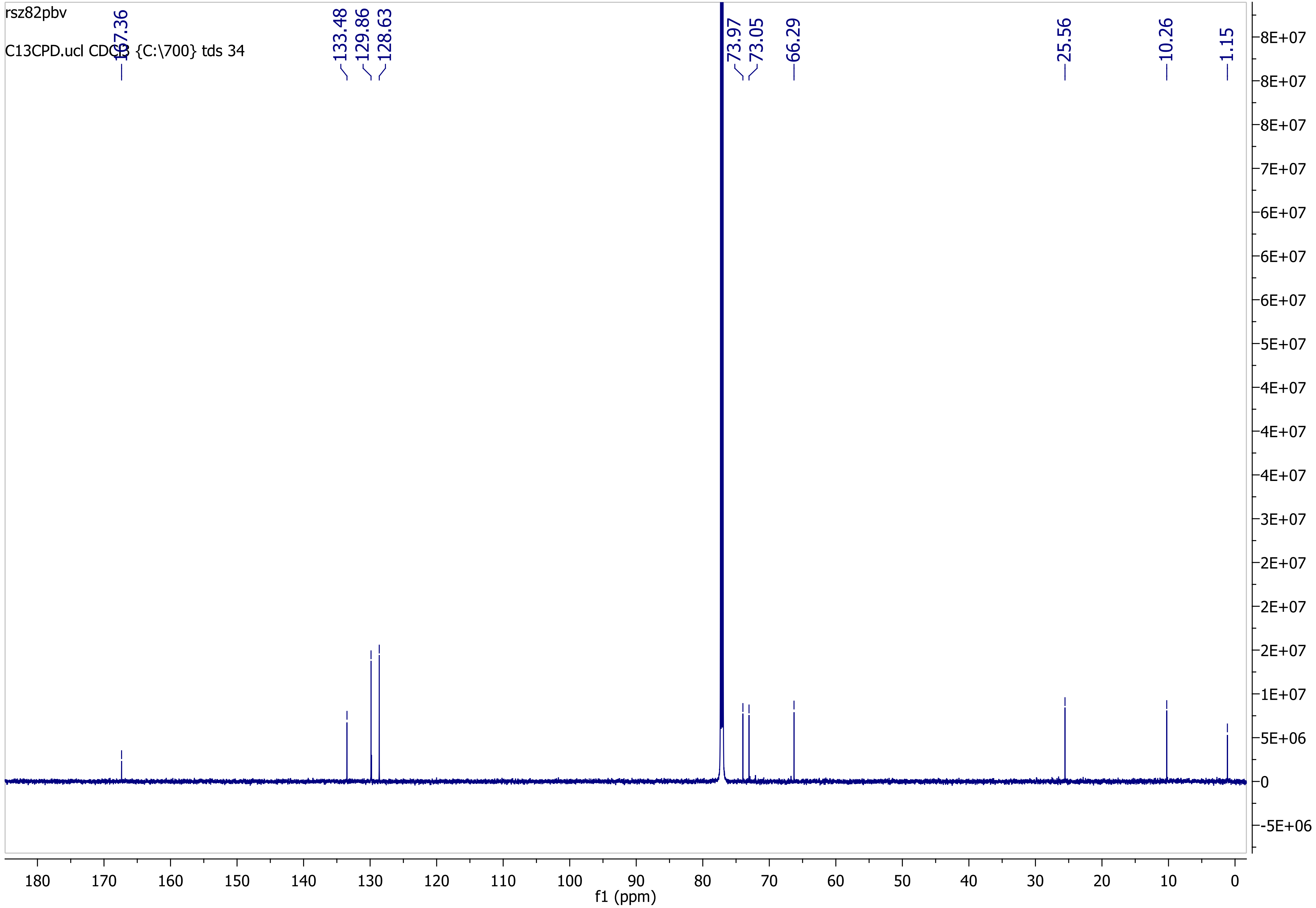
rsz82pbv
C13CPD.ucl CDCl3 {C:\700} tds 34
167.36
133.48
129.86
128.63
73.97
73.05
66.29
25.56
10.26
1.15
f1 (ppm)

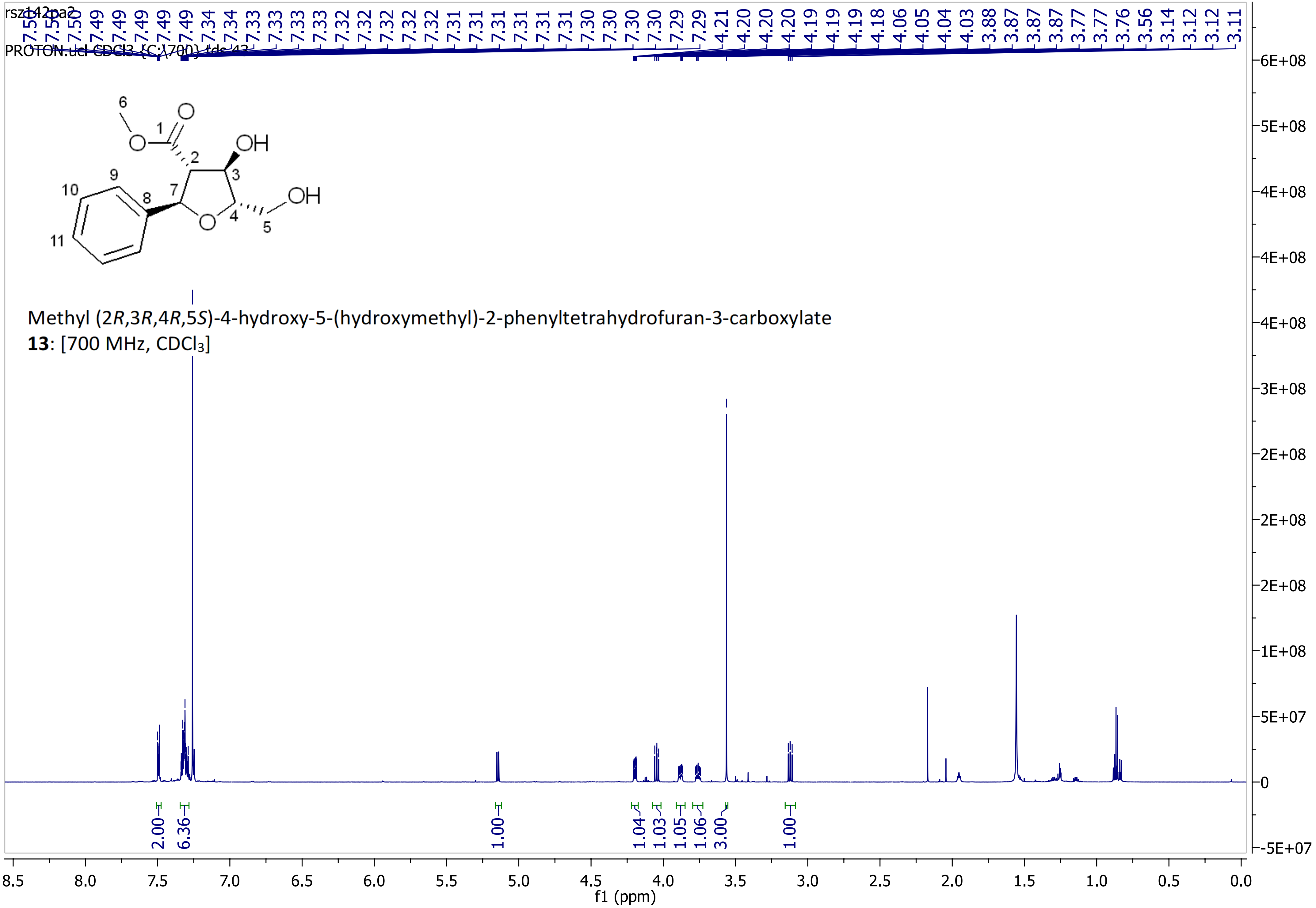
rsz142pa2
PROTON.ucl CDCl3 {C:\700} tds 43
7.50 7.50 7.50 7.49 7.49 7.49 7.49 7.49 7.34 7.34 7.33 7.33 7.33 7.33 7.32 7.32 7.32 7.32 7.32 7.31 7.31 7.31 7.31 7.31 7.31 7.30 7.30 7.30 7.30 7.29 7.29 4.21 4.20 4.20 4.20 4.19 4.19 4.19 4.18 4.06 4.05 4.04 4.03 3.88 3.87 3.87 3.87 3.77 3.77 3.76 3.56 3.14 3.12 3.12 3.11
Methyl (2*R*,3*R*,4*R*,5*S*)-4-hydroxy-5-(hydroxymethyl)-2-phenyltetrahydrofuran-3-carboxylate
**13**: [700 MHz, $CDCl_3$]
2.00
6.36
1.00
1.04
1.03
1.05
1.06
3.00
1.00
f1 (ppm)
8.5 8.0 7.5 7.0 6.5 6.0 5.5 5.0 4.5 4.0 3.5 3.0 2.5 2.0 1.5 1.0 0.5 0.0
6E+08 5E+08 4E+08 4E+08 4E+08 3E+08 2E+08 2E+08 2E+08 1E+08 5E+07 0 -5E+07
6
1
2
3
4
5
7
8
9
10
11
O
OH
OH
O
O

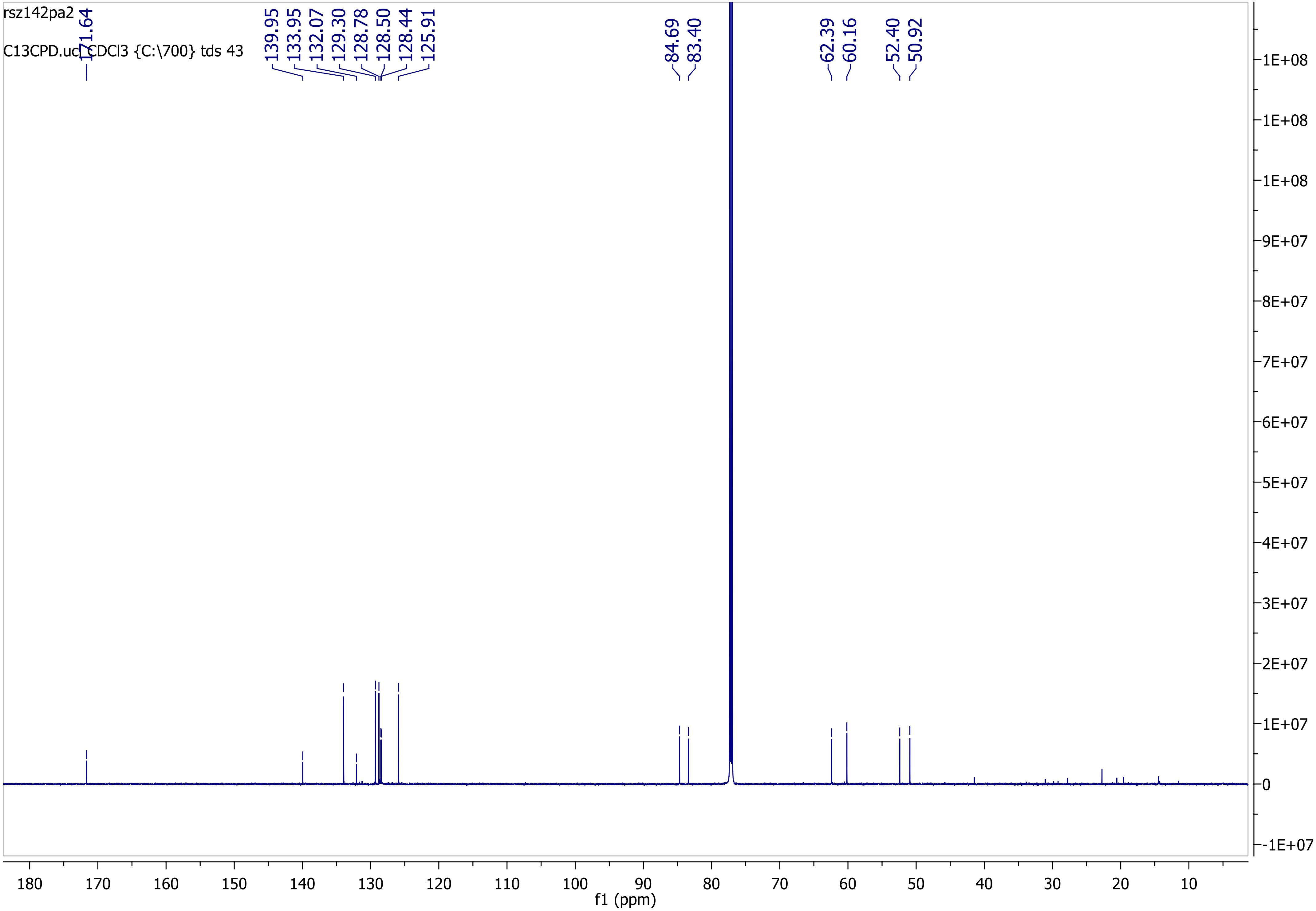
rsz142pa2
C13CPD.uc CDCl3 {C:\700} tds 43
171.64
139.95
133.95
132.07
129.30
128.78
128.50
128.44
125.91
84.69
83.40
62.39
60.16
52.40
50.92
f1 (ppm)

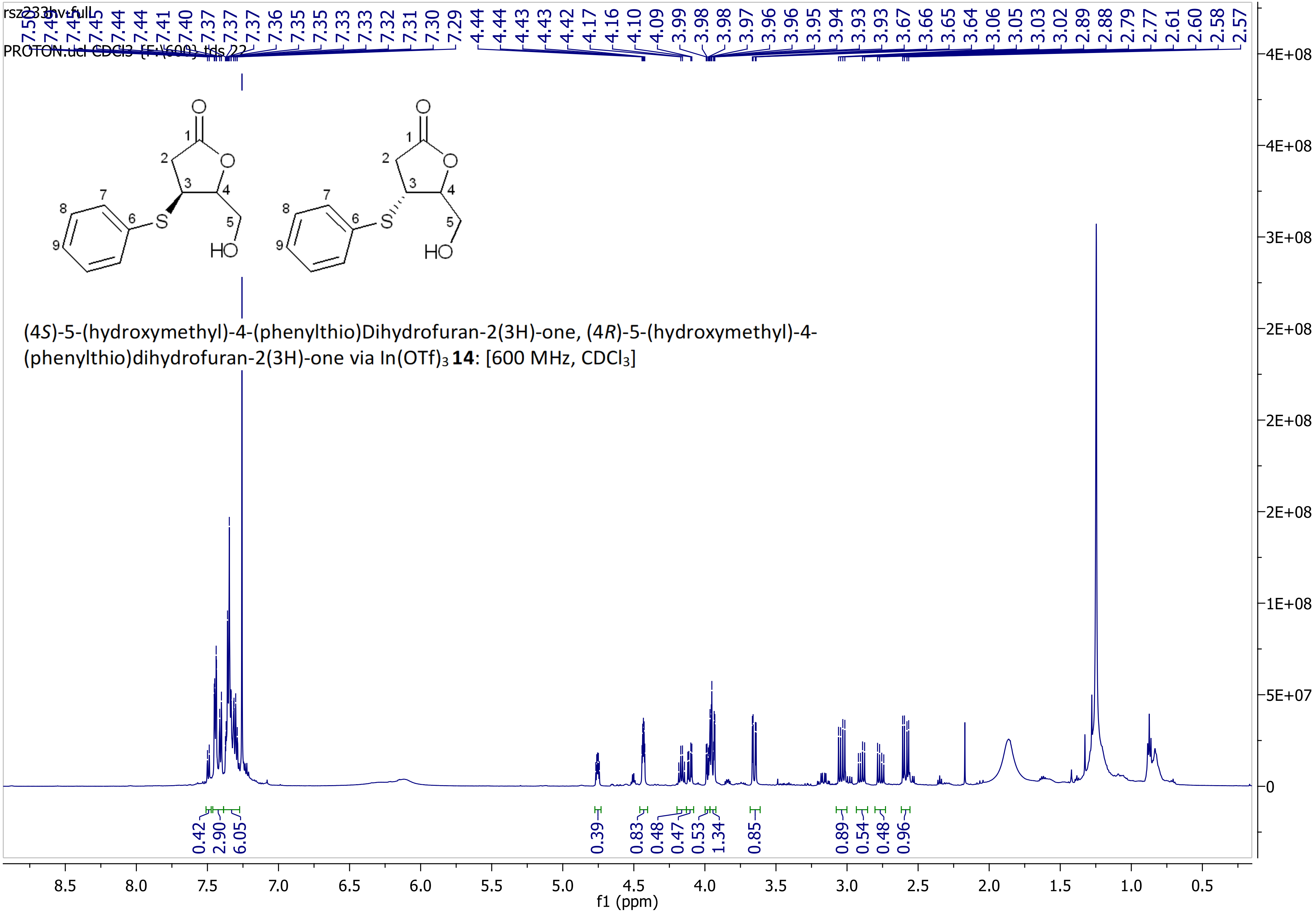


(4*S*)-5-(hydroxymethyl)-4-(phenylthio)Dihydrofuran-2(3H)-one, (4*R*)-5-(hydroxymethyl)-4-(phenylthio)dihydrofuran-2(3H)-one via $In(OTf)_3$ **14**: [600 MHz, $CDCl_3$]

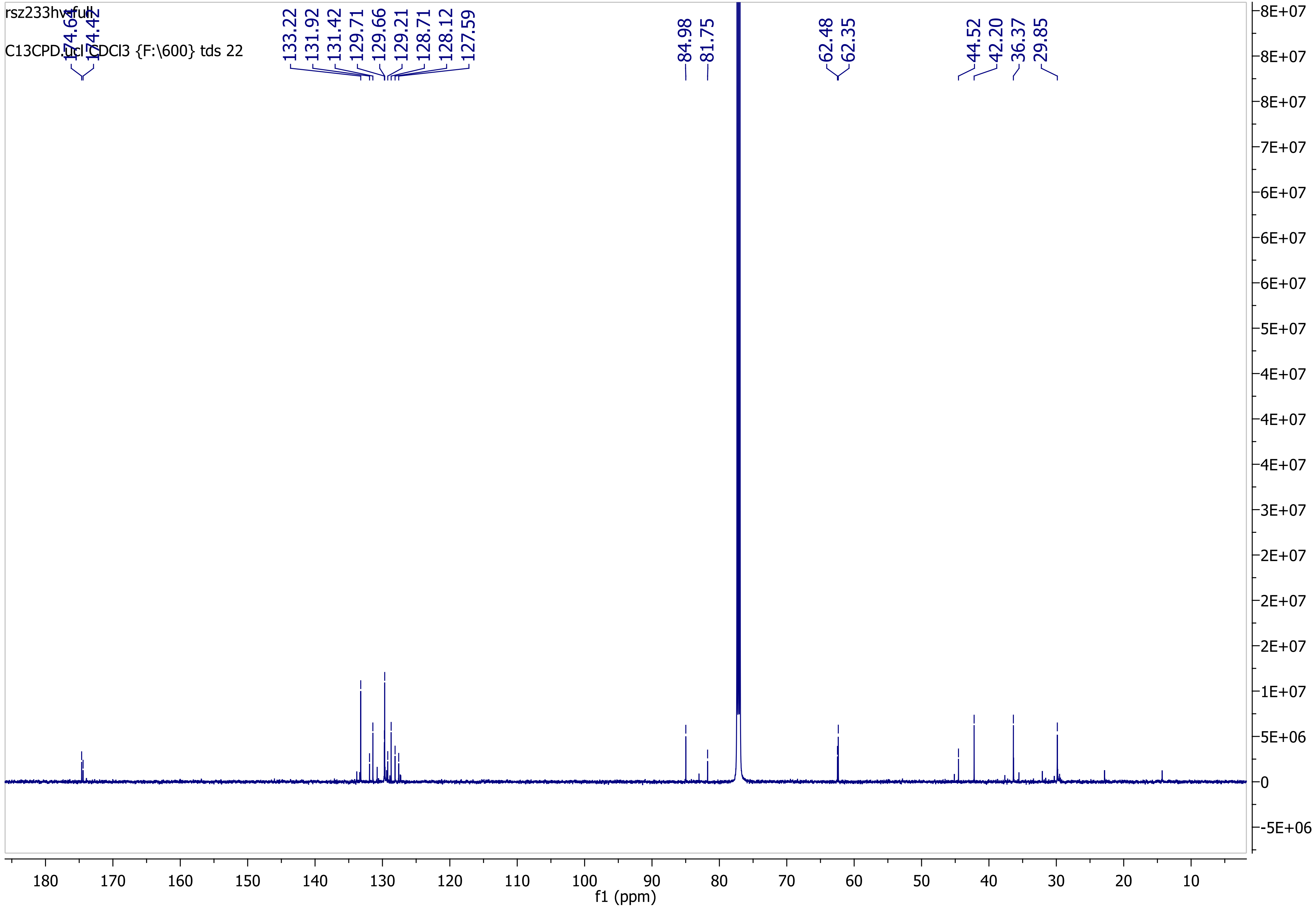

rsz233hv-full
C13CPD.ucl CDCl3 {F:\600} tds 22
174.61
174.42
133.22
131.92
131.42
129.71
129.66
129.21
128.71
128.12
127.59
84.98
81.75
62.48
62.35
44.52
42.20
36.37
29.85
8E+07
8E+07
8E+07
7E+07
6E+07
6E+07
6E+07
5E+07
4E+07
4E+07
4E+07
3E+07
2E+07
2E+07
2E+07
1E+07
5E+06
0
-5E+06
180 170 160 150 140 130 120 110 100 90 80 70 60 50 40 30 20 10
f1 (ppm)
